# Application of a GIS-Based Hydrological Model to Predict Surface Wetness of Blanket Bogs

**DOI:** 10.1007/s13157-023-01765-5

**Published:** 2024-01-03

**Authors:** Francis Mackin, Raymond Flynn, Fernando Fernandez-Valverde

**Affiliations:** 1 School of Natural and Built Environment, The Queen’s University of Belfast, David Keir Building, Stranmillis Road, Belfast, BT9 5AG UK; 2 National Parks and Wildlife Service, 90 King Street North, Smithfield, Dublin 7, Ireland

**Keywords:** Blanket bog, Hydrology, Ecohydrology, Topography, Modelling, Groundwater

## Abstract

Understanding hydrological processes operating on relatively intact blanket bogs provides a scientific basis for establishing achievable restoration targets for damaged sites. A GIS-based hydrological model, developed to assess restoration potential of Irish raised bogs, was adapted and applied to four relatively intact blanket bogs in Ireland. The Modified Flow Accumulation Capacity (MFAC) model utilised high-resolution topographic data to predict surface wetness, based on climatic conditions, contributing catchment and local surface slope. Modifications to MFAC parameters aimed to account for differences in hydrological processes between raised bogs and blanket bogs. Application of a climatic correction factor accounted for variations in effective rainfall between the four study sites, while monitoring of water table levels indicated a log-linear relationship between MFAC values and summer water table levels and range of water table fluctuations. Deviations from the observed relationship between MFAC and water table levels were associated with hydrological pressures, such as artificial drainage or the occurrence of subsurface macropores (peat pipes), which further lowered summer water tables. Despite being effective as a predictor of relative surface wetness, the relationship between MFAC and ecological variables such as *Sphagnum spp.* cover proved poor, pointing to the impact of past activities and damage caused by anthropogenic pressures. Findings demonstrated MFAC as an effective tool in predicting surface wetness within blanket bog-covered landscapes, thus proving useful to peatland practitioners in planning and prioritising areas for restoration.

**Supplementary Information:**

The online version contains supplementary material available at 10.1007/s13157-023-01765-5.

## Introduction

 Blanket bogs are a distinctive habitat found in high-latitude oceanic climates, including parts of Europe, North America, South America, Asia and Oceania (Gallego-Sala and Prentice 2013). Unlike many other wetland types, they develop on undulating topography and can form on relatively steep slopes (Lindsay 1995; Charman 2002). Despite covering a relatively small proportion of the global land surface, blanket bogs are the most common wetland type across Great Britain and Ireland, covering approximately 7.5% of Great Britain (Joint Nature Conservation Committee 2011) and 13% of the island of Ireland (Hammond 1981).

In recognition of their ecological importance, blanket bogs are listed for protection under Annex I of the European Union Habitats Directive (European Union Directive 92/43/EEC). Active blanket bogs that support significant areas of peat-forming vegetation are classified as a priority Annex I habitat type and are subject to strict protection. Habitat damaged by human activity requires restoration to peat-accumulating conditions (EU 2013). In addition to being an internationally important habitat, blanket bogs can also provide a wide range of ecosystem services, when maintained in a healthy ecological condition. This includes providing habitat to a range of specialist flora and fauna, providing a supporting function to downstream aquatic ecosystems (Flynn et al. 2021a; Kuemmerlen et al. 2022), and regulating river flow (Acreman and Holden 2013; Allott et al. 2019; Bain et al. 2011; Wilson et al. 2011).

Blanket bogs form a significant carbon store, and when maintained in a relatively intact state, can sequester carbon from the atmosphere (Roulet et al. 2007; Sottocornola and Kiely 2010; Creevy et al. 2020). In addition, across Great Britain and Ireland, blanket bog catchments act as an important source of drinking water due to high and frequent rainfall inputs, combined with low pollution pressures, ensuring a good raw water quality in large volumes (Parry et al. 2014; Xu et al. 2018). However, anthropogenic pressures can lead to degradation of the peatland habitats, diminishing raw water quality and resulting in increased water treatment costs. Water quality changes, due to peatland degradation, prove of particular concern due to elevated organic carbon loads and increased risk of disinfection by-products, such as trihalomethanes (THMs), exceeding water quality standards (Chow et al. 2003; O’Driscoll et al. 2018).

Historically, peatlands across Great Britain and Ireland have experienced impacts from a range of activities, including agriculture, afforestation, peat extraction and more recently wind energy development (Holden et al. 2007; Evans and Warburton 2011; Renou-Wilson et al. 2011). These activities not only result in the loss of an internationally important habitat, but also impact on a peatlands capacity to sustain ecosystem services. Changes to ecosystem services can include peatlands switching from a net carbon sink to a net carbon source (Wilson et al. 2015; Kritzler et al. 2016), loss of unique flora/fauna and impacts to water quality.

The importance of hydrological conditions, such as water table levels and range of water table fluctuations, in maintaining healthy ecological conditions in peatlands is well-established (Ingram 1983; Schouten 2002). It is generally recognised that peat-accumulating conditions require a stable and high-water table, close to the ground surface for peat to accumulate and sequester carbon (Nugent et al. 2018; Regan et al. 2020; Evans et al. 2021). Conversely their degradation results in reduced function, or even reversal of sequestration capacity, with degraded bogs releasing supplemental carbon to the atmosphere and water cycle. Many peatland restoration programmes attempt to reverse this damage, by raising the water table level closer to the ground surface and reducing the range of water table fluctuation, through measures such as drain blocking, removal of plantation forestry and bunding (Holden et al. 2008; Parry et al. 2014; Mackin et al. 2017a).

The success of restoration measures depends on a range of factors, such as depth of drainage, drain spacing, peat properties, peat thickness and restoration method. However, due to the close relationship between topographic parameters and hydrological conditions in peatlands, topography is considered a key factor in controlling restoration success (Graniero and Price 1999; van der Schaaf and Schouten 2002; Oosterwoud et al. 2017; Williamson et al. 2017; Crowley et al. 2021).

Damage to hydrological conditions on peatlands can be addressed by restoration measures including drain blockage on relatively flat peatlands, such as raised bogs. However, greater relief on blanket bogs makes restoration more challenging, with hydrological benefits of drain blocking becoming more limited with increasing slope. Moreover, relatively poor integrated characterisation of blanket bog hydrological processes, limits the confidence with which restoration targets may be established, and measures implemented to achieve them.

Cost-effective blanket bog restoration needs to be underpinned by scientific evidence to support decisions. By enhancing current understanding of ecohydrological processes (the interaction between ecological conditions and hydrology), peatland practitioners can develop more appropriate management and restoration strategies. In the case of Irish raised bogs, a significant body of research has been carried out to advance understanding of their ecohydrology (Schouten 2002; Regan et al. 2020). This includes the development of a comprehensive vegetation classification system involving the mapping of discrete ecotopes, in which various plant communities are associated with specific physical conditions, such as water table level and water chemistry (Kelly 1993; Fernandez et al. 2014; Cushnan 2018; Regan et al. 2020). While raised bogs and blanket bogs are typically considered to be largely isolated from interactions with underlying groundwater (Lindsay 1995), more recent studies have identified that these ecosystems can in some instances be groundwater dependent, relying on elevated heads in underlying substate to limit vertical losses of water to depth (Flynn et al. 2021a; Regan et al. 2019).

By comparing topographic and hydrological parameters, in combination with ecotope mapping, Mackin et al. (2017b) developed a hydrological model, based on the assumption that hydrological processes on raised bogs are dominated by surface/near-surface processes. The modelling protocol developed utilised high resolution topographic data collected using LiDAR (Light Ranging and Detection) data as a predictor of near-surface hydrological behaviour, in a similar way to the widely utilised topographic index (Beven and Kirkby 1979). The underpinning equation for the modelling process, based on modifying the Potential Acrotelm Capacity (PAC) equation, reported by van der Schaaf (2002) for a small number of raised bogs in the Irish Midlands, can be expressed as follows:

1 $$MFAC=\left(\frac{\sqrt{A}}{s}\right). K,$$

where:

*MFAC*: Modified flow accumulation capacity (km)
*A*: Upstream contributing catchment area (flow accumulation) (m^2^)
*S*: Local surface slope (m km^−1^)
*K*: Empirical climatic correction factor derived depending on rate of effective rainfall

MFAC values provide a proxy measurement of relative surface wetness, with higher MFAC values found to be associated with areas with a shallow water table and low MFAC values associated with areas with a deeper water table. Findings suggested that MFAC values of > 30 km in raised bogs corresponded closely to areas of peat-accumulating ecotopes, or degraded areas likely to return to peat-forming conditions through restoration. While the model was found to be effective where losses of water to the underlying substrate proved negligible, performance proved poorer in locations with significant water loss to depth.

Mackin et al. (2017b) expanded application of the MFAC model across the national network of designated raised bogs in Ireland, spanning a climatic gradient, from bogs receiving < 800 mm/yr rainfall in the east of Ireland, to bogs receiving > 1,200 mm/yr rainfall in the west, through the further development of the climatic correction factor. MFAC outputs indicated that with increasing rainfall, peat accumulating vegetation, dominated by *Sphagnum spp.*, can develop on steeper slopes in raised bogs. Although there are differences in hydrological processes between raised bogs and blanket bogs, hydrological conditions also underpin the development of peat-accumulating vegetation on blanket bogs (Lindsay 1995; Flynn et al. 2021b). Therefore, despite greater precipitation inputs and steeper slopes encountered on blanket bogs, the ability of the MFAC model to account for differences in precipitation inputs and surface slopes on raised bogs suggests that the processes considered by the MFAC model may also be applicable to blanket bog habitat. This provides a basis for better understanding blanket bog hydrology while also assisting in the restoration of hydrological damage to blanket bogs, which is typically focussed on measures to restore hydrological conditions (Armstrong et al. 2009; Holden et al. 2011).

Although use of models such as the topographic index have been developed and used to test relationships with water table levels in peatlands (e.g., Lane et al. 2004; Allott et al. 2009), these studies often focus on bogs that have been subject to significant levels of degradation. While Allott et al. (2009) reported correspondence between topographic index and water table levels at the Peak District in the UK, the authors reported poor correspondence between topographic index and water table levels in intact areas. Furthermore, while use of the topographic index provides an indication of relative surface wetness throughout a catchment, climatic conditions are not accounted for, meaning while comparisons within sites may be feasible, comparisons between sites are not possible.

Given the successful application of the MFAC model to raised bogs (Mackin et al. 2017b), this paper examines the applicability of the approach to blanket bogs. More specifically, this study aimed to establish whether a relationship exists between MFAC and water tables, as well as MFAC and ecological conditions. The authors hypothesise that MFAC can provide a prediction of surface wetness in relatively intact blanket bogs and therefore can be used to predict water table levels. Furthermore, based on conditions observed on Irish raised bogs, the authors hypothesise that MFAC will be related to broad ecological parameters. More specifically, areas with high MFAC values are anticipated to have higher cover of *Sphagnum spp.*, while cover and height of *Calluna vulgaris* is predicted to be lower.

## Methods

### Site Description

**Fig. 1:**
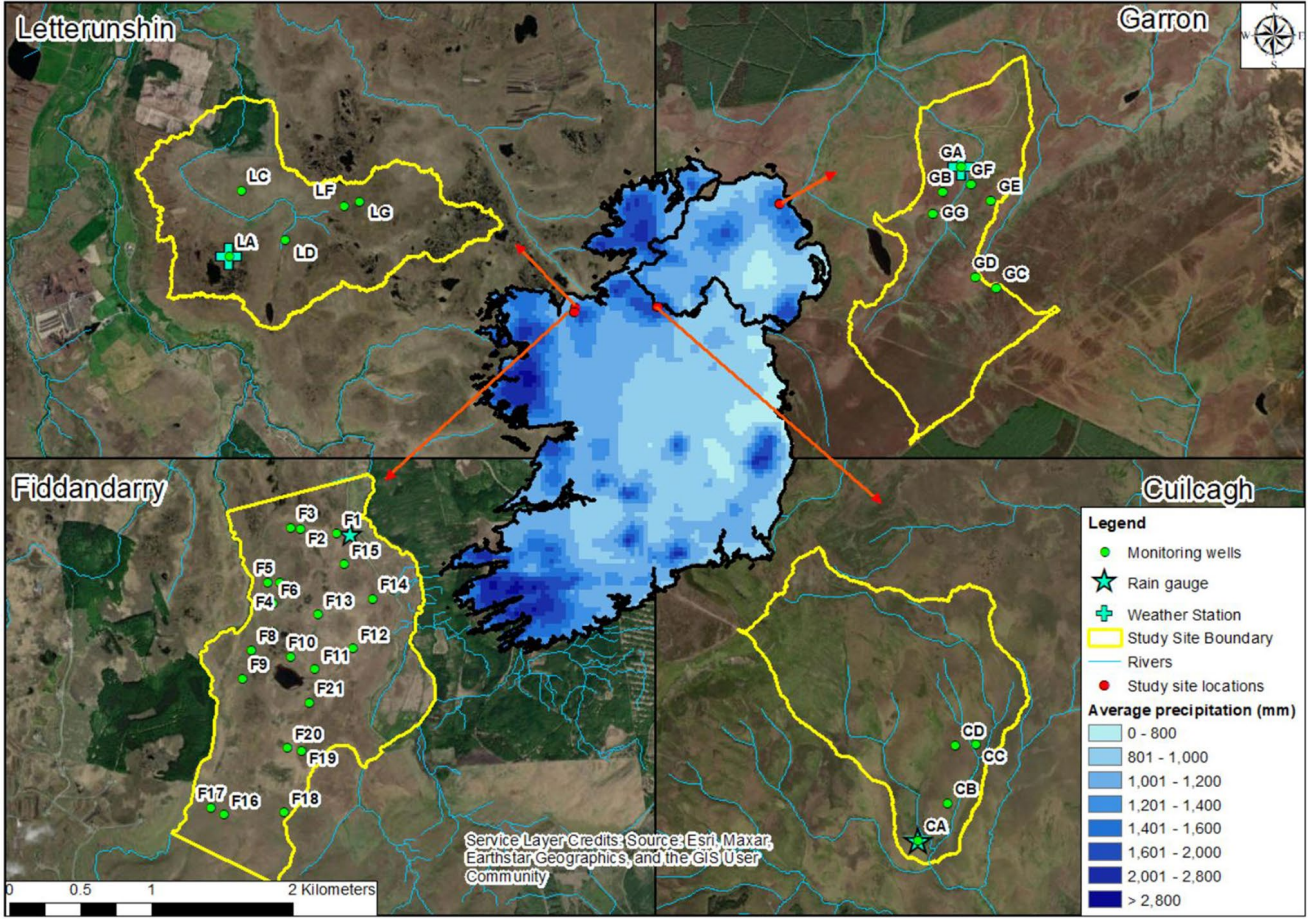
Location of the four study sites indicating average annual rainfall rate (Source: Met Éireann) along with locations of hydrological monitoring infrastructure (detailed individual site location maps provided as Figs. S1-S4 in Online Resource 1)

Four study areas, displaying almost complete coverage by relatively intact blanket bog, were selected spanning the east-west climatic gradient across the Island of Ireland. The four sites are Garron Bog, Co. Antrim, Cuilcagh Bog, Co. Cavan, Letterunshin Bog, Co. Sligo and Fiddandarry Bog, Co. Sligo (Fig. 1). All four sites occur within areas designated as Special Areas of Conservation (SACs), under the EU Habitats Directive, and have been selected due to the presence of habitats and species listed on Annex I and Annex II of the Directive, including the priority habitat blanket bog (if active). Garron, Cuilcagh and Letterunshin are drained by headwater streams, with site boundaries, illustrated in Fig. 1, relating to catchment boundaries, estimated using topographic data. Details of typical stream runoff rates for each of the catchment outlets are reported by Flynn et al. (2021a). In the case of Fiddandarry, the study site is divided by several sub-catchments and therefore the study site boundary is aligned to landownership boundaries.

The Garron study site (54.993^o^, -6.098^o^) (Garron) is a small headwater catchment, drained by the Collin Burn river, that forms part of the wider Garron Plateau SAC. The SAC contains the most extensive area of intact upland blanket bog in Northern Ireland (Joint Nature Conservation Committee 2022). Garron covers an area of 183 ha and has an elevation range of between 431.5 m above mean sea level (AMSL) and 278.5 mAMSL, with an estimated annual average rainfall rate of 1,694 mm, based on Met Éireann rainfall grids (Walsh 2012). Corine Land Cover (CLC) mapping identifies the entire catchment as peat bogs, while current land use involves low intensity grazing by sheep. The study site is dominated by upland blanket bog characterised by presence of *Calluna vulgaris* and *Eriophorum vaginatum* (Flynn et al. 2021b). Despite being one of the most intact areas of blanket bog in Northern Ireland, Garron has been subject to historical damage, due to pressures including overgrazing, drainage and burning (McKeown and Corbett 2017). However, over the past decade efforts to implement restoration measures and restore hydrological conditions across the site have included blocking of drainage ditches, reducing stocking density of sheep and preparation of a conservation action plan (NI Water 2019). Although there were some shallow drains blocked as part of restoration efforts in 2018/19 within the study site, artificial drainage is largely absent, with restoration programme efforts focussed on more degraded sections of the SAC.

The Cuilcagh study site (54.191^o^, -7.796 ^o^) (Cuilcagh) is located within the Cuilcagh–Anierin Uplands SAC in the Republic of Ireland. This SAC includes a series of uplands in counties Cavan and Leitrim, including Cuilcagh Mountain, which are contiguous to Cuilcagh Mountain SAC in Northern Ireland. The study catchment covers an area of 239 ha. It has an elevation range of between 660 mAMSL to 300 mAMSL, at the catchment outlet, with estimated average annual rainfall of 2,200 mm (Walsh 2012). CLC mapping identifies the entire catchment as peat bogs, while current land use involves low-intensity grazing by sheep. The study site is located towards the north of the SAC in an area incised by a series of steeply sloping streams that converge and form the headwaters of the Cladagh-Swanlinbar River. The vegetation on the study site is primarily upland blanket bog, dominated by *Calluna vulgaris* and *Eriophorum vaginatum*, along with areas of poor fen and flush with *Juncus* and *Sphagnum* species (Perrin et al. 2013a). Parts of the site display evidence of burning within the past decade, while no ostensible evidence of artificial drainage exists.

The Letterunshin study site (54.185 ^o^, -8.911 ^o^) (Letterunshin) and Fiddandarry study site (54.149 ^o^, -8.927 ^o^) (Fiddandarry) both occur within the Ox Mountains Bogs SAC in Co. Sligo. The SAC contains extensive areas of active blanket bog, along with dystrophic bog pool systems and quaking lawns, characterised by presence of *Sphagnum cuspidatum* and *Rhynchospora alba* (NPWS 2016).

Letterunshin has an elevation ranging between 149 mAMSL and 107 mAMSL and is drained by the Fiddanduff River. CLC mapping identifies the entire catchment as peat bogs with a small section at the downstream end of the catchment being planted with coniferous forestry. Current land use involves low-intensity grazing by sheep along with domestic scale peat extraction close to the western margins of the catchment. Letterunshin has an average annual rainfall rate of 1,465 mm (Walsh 2012) and consists of a relatively flat expanse of lowland blanket bog, dominated by *Calluna vulgaris*, *Eriophorum angustifolium*, *Eriophorum vaginatum*, *Narthecium ossifragum* and various *Sphagnum* species, along with several areas dominated by Rhynchosporion vegetation (Perrin et al. 2013b). The site contains a series of dystrophic pool systems on the flatter slopes. A network of spontaneously developed peat pipes, evident through the occurrence of vertical shafts/swallow holes features in the bog surface, discharge at the ground surface to form the source of the Fiddanduff river (Flynn et al. 2022). While there is evidence of burning in the past and some small-scale peat extraction close to the margins, the remainder of the site lacks artificial drainage and remains in a relatively intact condition (Perrin et al. 2013b).

Flynn et al. (2021b) reported the presence of submerged peat piping at Cuilcagh, Garron and Letterunshin, despite the absence of ostensible disturbance to the natural drainage regime. Although some of these features had visible topographic expression, elsewhere their identification proved more elusive, in some cases being identified only by audible cascading water, following intense rainfall.

Measurements undertaken by Flynn et al. (2022) at Letterunshin provided estimates of peat hydraulic conductivity ranging from 10^0^ m/day to 10^−2^ m/day in peat within 1 m of the ground surface at locations away from known peat pipes, where peat humification typically ranged between H1 and H4 in the von Post system (von Post 1922). Hydraulic conductivities declined by up to 2 orders of magnitude at the base of the peat, where humification increased to H7-H8, and by a similar magnitude approaching peat pipes. Flynn et al. (2021b) reported comparable trends at Garron and Cuilcagh.

Fiddandarry, located c. 2 km south of Letterunshin, covers an area of 296 ha and has elevations ranging between 170 mAMSL to 110 mAMSL, with an average annual rainfall rate of 1,455 mm (Walsh 2012). CLC mapping identifies the entire catchment as peat bogs, while current land use involves low-intensity grazing by sheep. Habitat at Fiddandarry is a transition from lowland to upland blanket bog. The study site is relatively flat with the ground surface rising rapidly to the south. The study site contains parts of two catchments, with the western portion of the site draining towards the Gowlan River and the eastern portion of the site draining towards the Owenwee River, both of which are tributaries of the River Easky. The vegetation at Fiddandarry resembles that of Letterunshin, with the site dominated by lowland blanket bog, along with large areas of Rhynchosporion vegetation and a complex of dystrophic pools (Perrin et al. 2013b). While there are large areas of relatively intact vegetation across the site, more damaged sections of bog are associated with a series of shallow artificial drainage ditches (typically 0.5–0.75 m deep), which were installed across the drier areas of Fiddandarry during the 1980s (Douglas et al. 1989). These drains were blocked with peat dams in early 2021.

### Hydrological Monitoring

#### Water Level Monitoring

Monitoring wells, located in contrasting topographic settings, were installed across seven locations at Garron, four locations at Cuilcagh and five locations at Letterunshin (Fig. 1). Monitoring locations were selected based on analysis of 5 m resolution topographic data and aimed to monitor conditions in a range of contrasting hydrological settings. At each of the monitoring locations, three 1.5 m long, 32 mmID phreatic monitoring wells (monitoring wells), with 1.0 m long screened intervals, were installed in an approximately equilateral triangular array with ~ 7 m sides, following hand coring with a 20 mm diameter gouge auger to create a pilot hole. Where peat thickness exceeded 2 m, a piezometer, with 0.5 m screened interval, was installed at the centre of the triangular monitoring well array down to the base of peat. Persistent damage to equipment by livestock at Cuilcagh prevented installation of piezometers at this site. Data from monitoring wells and piezometers permitted determination of vertical and horizontal hydraulic gradients.

Twenty-one 1.5 m long, 32 mmID phreatic monitoring wells with 1.0 m long screened intervals, were installed across the Fiddandarry study site to monitor water levels under contrasting topographic conditions (Fig. 1). Ten monitoring locations also had a piezometer with a 0.5 m screen installed extending upwards from the base of peat, enabling monitoring of vertical hydraulic gradients. Piezometers were installed within 1 m of monitoring wells at Fiddandarry. Each water table monitoring location, across the four study sites, were instrumented with a Solinst Junior Edge levelogger® (5 m range; accuracy ± 0.1% or 0.5 cm) (Solinst, ON) to record water table levels at hourly intervals, while piezometers were measured manually. All monitoring wells and piezometers were sealed at the base, while a cap was placed on each well to prevent direct ingress of precipitation. One Solinst Barologger® (accuracy ± 0.1%) was installed on each study site to measure site-specific atmospheric pressure and enable barometric correction of water levels.

Hourly measurements of water table levels were recorded throughout from mid-2017 – August 2020 at Garron, from August 2017 – April 2021 at Letterunshin, from October 2017 – November 2019 at Cuilcagh and from March 2019 – September 2020 at Fiddandarry. Manual dipping at approximately quarterly intervals was carried out to validate automated readings and to provide supplemental water level data from monitoring wells and piezometers lacking automated water level loggers.

Measurements of groundwater levels from fixed points at the top of monitoring well casing, minus the distance to the ground surface permitted measurement of the water table depth and groundwater head at the base of the peat. Water table levels are reported in metres relative to ground surface, with negative values indicating depth below ground surface and positive values indicating height above ground level.

Using water level logger data, water table statistics were calculated for summer periods (defined as April-September) when the most significant declines in water table levels occur, including:

- D90 = water table level, measured in metres relative to ground surface, that is equalled or exceeded (i.e., shallower) for 90% of the monitoring period (calculated as the 10th percentile of hourly summer water table measurements).
- Median = water table equalled or exceeded for 50% of the monitoring period (calculated as the 50th percentile of hourly summer water table measurements).
- D10 = water table level equalled or exceeded for 10% of the monitoring period (calculated as the 90th percentile of hourly summer water table measurements).
- D1 minus D99 (water table fluctuations) = D1 (water table level water table level equalled or exceeded for 1% of the monitoring period [calculated as the 99th percentile of hourly summer water table measurements]) minus D99 (water table level water table level equalled or exceeded for 99% of the monitoring period [calculated as the 1st percentile of hourly summer water table measurements]).

#### Meteorological Conditions

At Garron and Letterunshin, a Davis Instruments Vantage Pro-plus Weather Stations (Davis Instruments, CA) enabled measurements of precipitation and automated calculation potential evapotranspiration (PE) at 30-minute intervals calculated using a modified Penman-Monteith method (Allen et al. 1998). Automatic tipping bucket rain gauges at Cuilcagh (Onset Hobo (Onset Instruments, MA) and Fiddandarry (Solinst RG1 (Solinst, ON) measured precipitation at these sites. Instrument failure at Fiddandarry resulted in precipitation records being supplemented with data from Letterunshin.

### LiDAR

Airborne LiDAR (Light Ranging and Detection) surveys were carried out in 2017 by Bluesky International using an Optech Galaxy LiDAR Sensor. Minimum point density was 8ppm and estimated vertical accuracy of +/- 0.15 m. A high resolution (1 m) Digital Terrain Model (DTM) (i.e., bare-earth model) was provided in ASCII format. This resolution has been found to be adequate for ecohydrological modelling of peatlands in Ireland in previous studies (Mackin et al. 2017b; Regan et al. 2019, 2020).

### GIS-Based Modelling

The hydrological model, developed by Mackin et al. (2017b) for Irish raised bogs, was adapted for application to each of the study sites using ESRI ArcMap 10.8®. Briefly, the Mackin et al. (2017b) model involved filling sinks in the DTM grid, identifying flow direction using the D8 algorithm (Greenlee 1987), determining flow accumulation and defining flow patterns across the bog surface. Contributing catchment area was then calculated at 5 m intervals along each of the defined flowlines and flow accumulation interpolated across the catchment area using the natural neighbour method (Sibson 1981). Surface slope was calculated with spatial analyst tools within ArcMap, employing a modified version of the DTM grid to eliminate the confounding effects of microtopography on local surface slope. This involved smoothing the 1 m DTM using the Focal Statistics Tool in ArcMap, to provide an average elevation over an area of 20 m. Incorporation of site-specific climatic correction factors permitted calculation of MFAC (Eq. 1) using Map Algebra tools (ESRI 2022).

Subsequent adaptations for application to blanket bog included smoothing DTM cell size to 5 m; the original 20 m cell resolution was considered inappropriate, given the potential for more significant changes in elevation over a 20 m distance on blanket bogs compared to raised bogs. The climatic correction factor (K) was based on estimates of long-term average rates of effective rainfall at each of the project sites, with precipitation derived from Walsh (2012); interpolation of rates of PE reported at Met Éireann and Met Office synoptic weather stations provided an estimate of evapotranspiration rates. For the four blanket bog sites, the K factor was determined by dividing the annual average effective rainfall rate for each site by the average annual effective rainfall rate for Letterunshin to allow results for other sites to be scaled relative to Letterunshin (Table 1).

**Table 1:** Climatic correction factor and stream formation threshold applied to each study site

Study site	Estimate of annual average effective rainfall (mm)	Climatic K factor	Stream formation threshold (ha)
Garron	1,194	1.32	2.0
Cuilcagh	1,586	1.75	1.5
Letterunshin	907	1	5.0
Fiddandarry	900	0.99	5.0

The Mackin et al. (2017b) MFAC model assumes that surface wetness increases with larger contributing catchment area and shallower local surface slope. However, this relationship breaks down when contributing catchment area results in formation of a defined drainage channel. In contrast to the assumptions of the Mackin et al. (2017b) MFAC model predictions, formation of drainage channels results in a drainage effect on the peat, thus reducing surface wetness. Consequently, initial MFAC outputs were highly skewed by the occurrence of natural watercourses and depressions formed above large peat pipes. Accordingly, modifications to estimates of contributing catchment area, (parameter *A*, in Eq. 1) proved necessary. Drains and large peat pipes were excluded on a site-specific basis by setting a threshold value for *A*, above which stream formation occurred or where peat pipes were apparent from topographic conditions.

Due to the variation in climatic conditions and topography across the four study sites, it was necessary to define an empirical stream formation threshold for each study site. This involved reviewing contributing catchment area and undertaking a visual comparison of aerial imagery and topographic conditions, as illustrated by LiDAR-derived DTMs. Analysis involved identifying where surface drainage first occurs and determining minimum catchment area thresholds required to form such features. The stream formation threshold determined varied across the four sites, with the lowest stream formation threshold identified at the Cuilcagh study site. A higher stream formation threshold was identified for Garron, with the highest thresholds identified at Letterunshin and Fiddandarry, as outlined in Table 1.

### Ecological Monitoring Plots

**Fig. 2:**
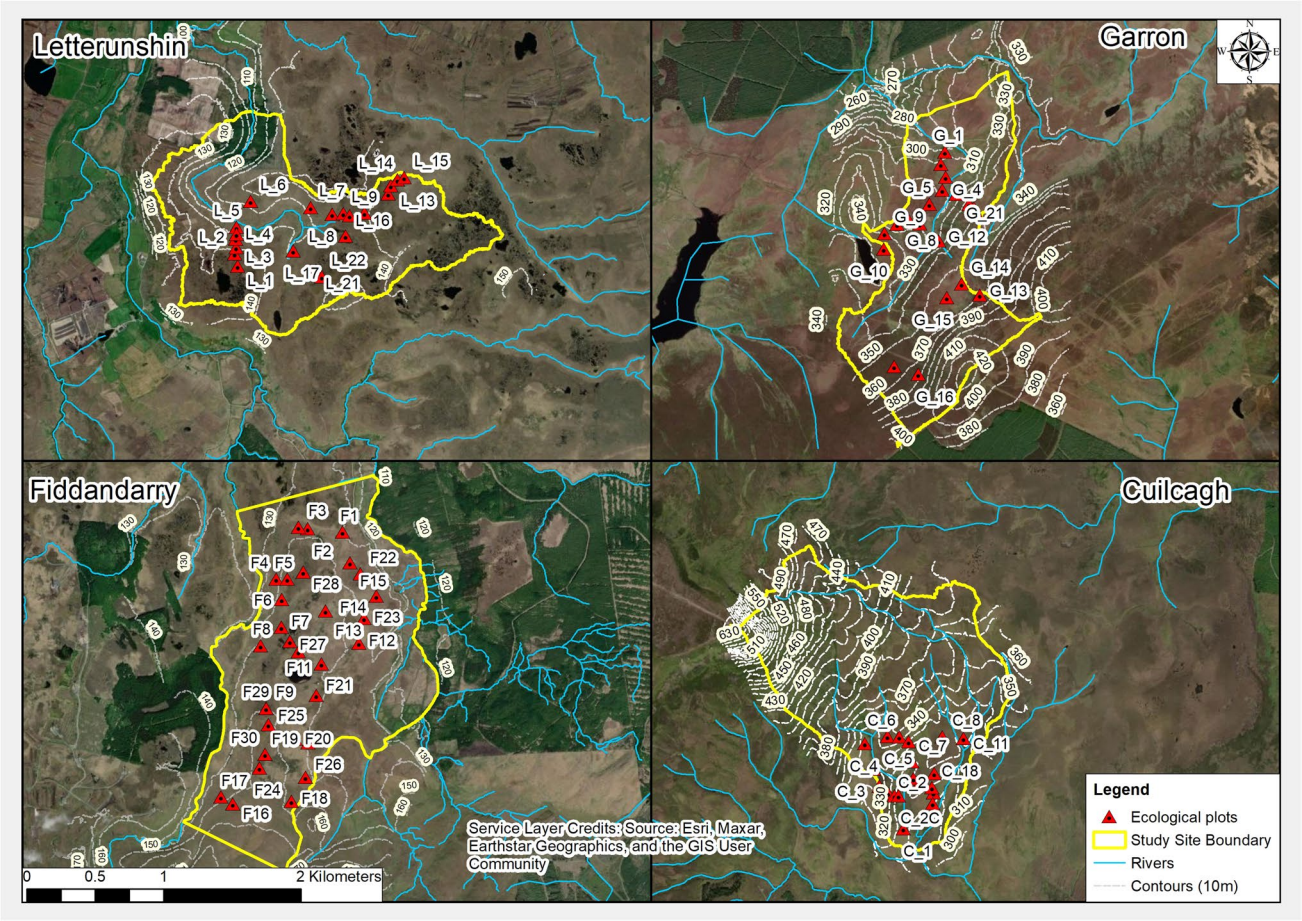
Location of ecological monitoring plots at each of the four study sites, with 10 m contours presented to illustrate topographic conditions

**Fig. 3:**
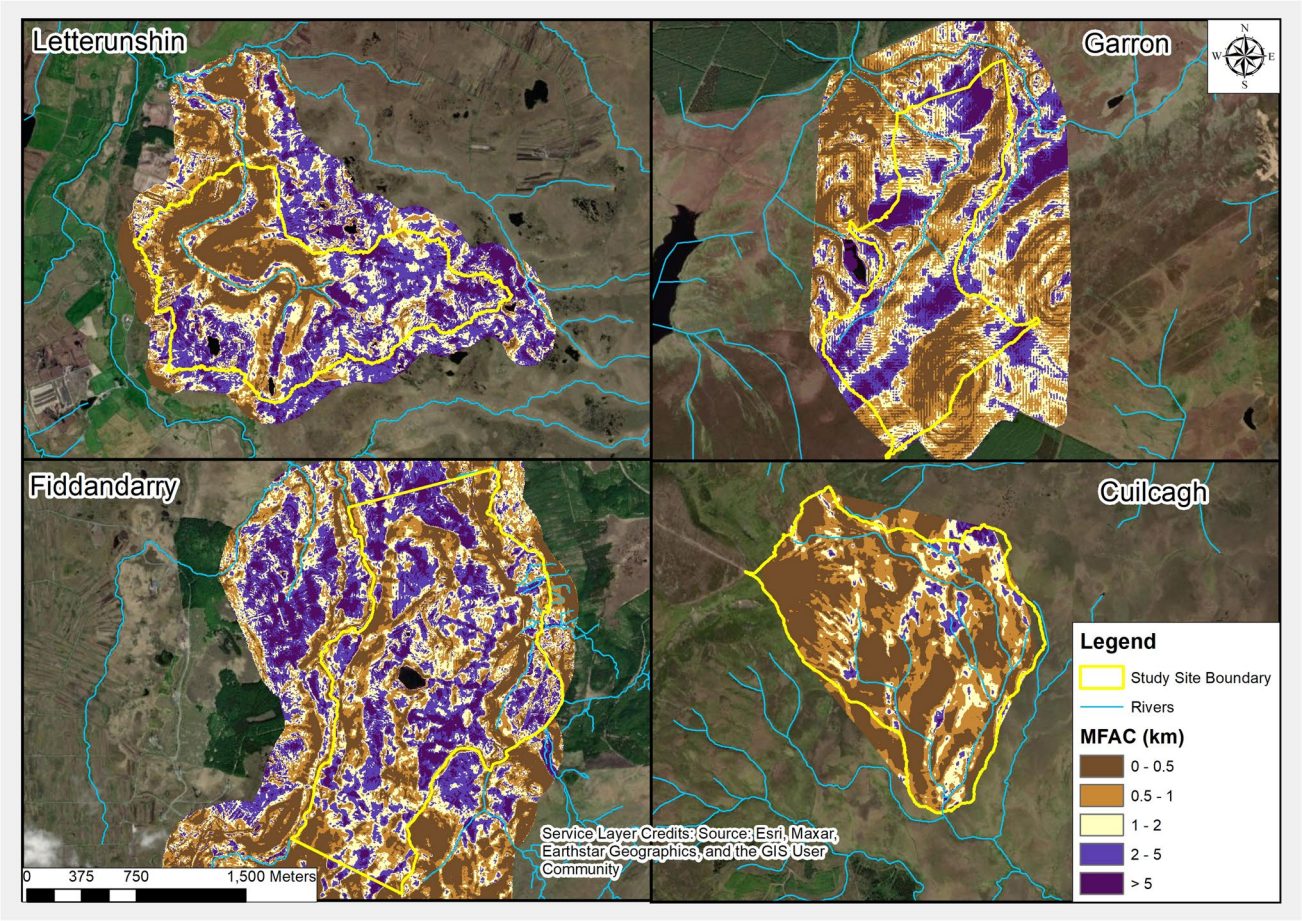
Modified Flow Accumulation Capacity (MFAC) at the four study sites

To test the relationship between MFAC and ecological conditions, field-based ecological monitoring plots were carried out at each of the study sites. Ecological assessments aimed to use rapid assessments of broad ecological variables and compare these with MFAC values to determine whether a relationship could be established. These assessments focused on cover of *Sphagnum* spp., *Calluna vulgaris*, *Molinia caerulea*, bare peat and depth to humification level H4 on the von Post scale (von Post 1922) (see Table S4 in Online Resource 1 for full details of ecological parameters recorded). A total of 90 monitoring plots, each measuring 4 m x 4 m, were recorded (19 at Cuilcagh, 21 at Garron, 20 at Letterunshin and 30 at Fiddandarry) across variable topographic settings (Figs. 2 and 3). Monitoring plots aimed to capture the variation in conditions across each of the study sites and were targeted to provide adequate coverage across the dominant vegetation types on each site rather than using a random sampling method.

Parameters were adapted from the approach taken in assessing the condition of Irish raised bogs, as outlined by Fernandez et al. (2014), whereby there is a strong relationship between *Sphagnum* spp. cover and occurrence of peat-accumulating vegetation types. Peat probing was also carried out at each of the ecological monitoring plots. Peat probing involved using a peat probing kit to probe to the base of peat and recording peat thickness to the nearest 5 cm along with GPS coordinates using a Garmin etrex® 30 handheld GPS (approximately 2–3 m horizontal accuracy). In a sub-set of locations, where peat thickness exceeded 3 m, a 1.3 m long Eijkelkamp 20 mm gouge auger with 1 m length extendable rods was used to confirm peat thickness and record substrate type. Depth to H4 was determined by hand excavating a trial pit at each ecological monitoring plot and measuring the depth from ground surface to approximate boundary of humification level H4.

### Statistical Analysis

Relationships between MFAC and water table levels (D90 and D1 minus D99) and MFAC and ecological parameters, were assessed using regression analysis. In each case the coefficient of determination (r^2^) was reported, and significance of regression relationships tested using ANOVA (significance determined at the *P* < 0.05 and *P* < 0.01 level).

## Results

### Meteorological Conditions

Meteorological data collected during the monitoring period revealed less precipitation fell during summer months (April-September) compared to winter months (October-March), with fewer rain days (≥ 1 mm precipitation) and wet days (≥ 5 mm precipitation) during summer months (except for 2019 at Garron) (Table 2) (monthly meteorological conditions are provided in Table S1 in Online Resource 1). Weather stations at Garron and Letterunshin indicate low rates of PE throughout the winter months (October-March), with approximately 80% of annual PE reported during summer months (April – September). Overall, meteorological monitoring highlights that summer 2020 was notably drier than summer 2019 at all monitoring sites, with lower rates of precipitation and higher rates of PE.

**Table 2:** Summary of results from meteorological monitoring at each of the study sites

Garron	Year	Summer	Winter	Annual	Proportion Summer (%)	Proportion Winter (%)
Precipitation (mm)	2019	564.6	703.5	1268.1	45	55
PE (mm)		393.8	108.6	502.4	78	22
Rain days (≥ 1 mm)		93	103	196	47	53
Wet days (≥ 5 mm)		51	48	99	52	48
Precipitation (mm)	2020	545.0	757.6	1302.6	42	58
PE (mm)		405.0	98.6	503.6	80	20
Rain days (≥ 1 mm)		80	116	196	41	59
Wet days (≥ 5 mm)		39	60	99	39	61
Letterunshin	Year					
Precipitation (mm)	2019	718.9	808.0	1526.9	47	53
PE (mm)		471.8	127.3	599.1	79	21
Rain days (≥ 1 mm)		107	117	224	48	52
Wet days (≥ 5 mm)		54	64	118	46	54
Precipitation (mm)	2020	511.3	1159.5	1670.8	31	69
PE (mm)		520.4	153.3	673.7	77	23
Rain days (≥ 1 mm)		89	136	225	40	60
Wet days (≥ 5 mm)		35	83	118	30	70
Fiddandarry*	Year					
Precipitation (mm)	2019	718.9	783.6	1502.5	48	52
Rain days (≥ 1 mm)		102	111	213	48	52
Wet days (≥ 5 mm)		51	63	114	45	55
Precipitation (mm)	2020	523.4	1111.8	1635.2	32	68
Rain days (≥ 1 mm)		86	131	217	40	60
Wet days (≥ 5 mm)		33	76	109	30	70
Cuilcagh	Year					
Precipitation (mm)	2019	875.2	1359.8	2235	39	61
Rain days (≥ 1 mm)		92	124	216	43	57
Wet days (≥ 5 mm)		55	80	135	41	59

Proportion Summer (%) Summer and Winter (%) indicates proportion of totals occurring within summer (April-September) and winter (January -March & October-December) periods

*Note: missing records at Fiddandarry were supplemented by records at Letterunshin

### Water Table Levels and Hydraulic Gradients

Monitoring data suggested that during winter periods (October - March), other than at one monitoring well, winter D90 water table levels remained within 20 cm of the ground surface at all monitoring locations. In contrast, water table levels, proved more variable during summer months, with summer D90 levels as low as 54 cm below ground level recorded.

Horizontal hydraulic gradients closely reflect topographic conditions, with a strong, significant relationship between horizontal hydraulic gradients reported and local surface slope (r^2^ = 0.95; F_1,14_ = 292.63, *P* < 0.001). Steeper horizontal hydraulic gradients were recorded at monitoring wells on steep slopes. The lowest minimum and maximum horizontal hydraulic gradients were recorded at nest LA (slope = 0.6%), with the highest minimum and maximum horizontal hydraulic gradients recorded at nest GC (slope = 13.5%). Vertical hydraulic gradients were generally downwards across all sites, with a small number of temporary slight upward gradients reported at nests GG, LA, LG, F19 and F20. Steepest downward vertical gradients were recorded at nests GB, GA and F1.

Table 3 provides a summary of summer median, summer D90 and summer water table fluctuations (D1 minus D99) for 2019 and 2020 along with ranges of horizontal and vertical hydraulic gradients from manual measurements (water level duration curves for summer and winter monitoring periods provided as Fig. S5, while horizontal and vertical hydraulic gradients are presented in Tables S2 and S3 in Online Resource 1).

**Table 3:** Summary of summer median, D90 and water table fluctuations (D1 minus D99) along with ranges of horizontal and vertical hydraulic gradients where piezometers are also installed

Well ID	MFAC (km)	Median - Summer 2019 (m)	Median - Summer 2020 (m)	D90 - Summer 2019 (m)	D90 – Summer 2020 (m)	D1 minus D99 - Summer 2019 (m)	D1 minus D99 - Summer 2020 (m)	Horizontal hydraulic gradient (range)	Vertical hydraulic gradient (range)
GA	3.47	-0.08	-0.09	-0.13	-0.2	0.15	0.26	0.028–0.054	0.310–0.438
GB	6.71	-0.06	-0.07	-0.1	-0.18	0.12	0.21	0.027–0.029	0.002–0.774
GC	1.17	-0.11	-0.14	-0.2	-0.28	0.17	0.25	0.158–0.176	-
GD	5.09	-0.05	-0.1	-0.15	-0.21	0.2	0.26	0.015–0.018	0.066–0.171
GE	3.79	-0.03	-0.04	-0.1	-0.2	0.17	0.24	0.012–0.026	0.019–0.368
GF	1.07	-0.1	-0.15	-0.22	-0.25	0.21	0.26	0.126–0.142	-
GG	3.92	-	-0.09	-	-0.16	-	0.16	0.022–0.060	-0.010–0.313
LA	4.96	-0.06	-0.08	-0.17	-0.23	0.22	0.3	0.008–0.016	-0.010–0.263
LC	0.4	-0.08	-0.11	-0.27	-0.31	0.34	0.36	0.095–0.096	-
LD	3.38	-0.02	-0.05	-0.12	-0.2	0.21	0.26	0.012–0.019	0.050–0.153
LF	1.21	-0.13	-0.17	-0.22	-0.31	0.18	0.32	0.039–0.042	0.048–0.153
LG	4.6	-	-0.07	-	-0.2	-	0.21	0.018–0.020	-0.005–0.044
CA	0.63	-0.15	-	-0.32	-	0.35	-	0.089–0.132	-
CB	1.61	-0.07	-	-0.17	-	0.23	-	0.029–0.058	-
CC	1.71	-0.07	-	-0.14	-	0.2	-	0.045–0.061	-
CD	2.51	-0.08	-	-0.18	-	0.2	-	0.022–0.028	-
F1	10.08	-0.05	-0.06	-0.1	-0.21	0.21	0.35	-	0.273–0.427
F2	0.36	-0.2	-0.21	-0.49	-0.54	0.57	0.61	-	-
F3	0.84	-0.08	-0.08	-0.17	-0.23	0.17	0.27	-	-
F4	0.46	-0.09	-0.17	-0.29	-0.44	0.32	0.48	-	-
F5	6.23	-0.02	-0.07	-0.09	-0.15	0.15	0.19	-	0.000–0.047
F6	4.54	-0.05	-0.05	-0.16	-0.2	0.21	0.26	-	-
F7	4.85	-0.05	-0.1	-0.18	-0.24	0.23	0.31	-	0.000–0.186
F8	0.23	-0.36	-0.28	-0.49	-0.53	0.51	0.57	-	-
F9	0.8	-0.07	-0.11	-0.22	-0.3	0.29	0.38	-	-
F10	2.11	-0.11	-0.14	-0.2	-0.25	0.2	0.27	-	0.006–0.043
F11	1.55	-0.05	-0.08	-0.14	-0.21	0.17	0.29	-	-
F12	3.08	-0.13	-0.16	-0.24	-0.28	0.3	0.33	-	0.033–0.187
F13	0.96	-0.12	-0.14	-0.21	-0.27	0.22	0.32	-	-
F14	4.85	-0.09	-0.1	-0.21	-0.26	0.22	0.31	-	0.000–0.067
F15	4.43	-0.06	-0.05	-0.12	-0.13	0.2	0.23	-	0.016–0.105
F16	8.63	-0.08	-0.11	-0.16	-0.2	0.2	0.23	-	-
F17	0.88	-0.12	-0.17	-0.25	-0.39	0.29	0.43	-	-
F18	4.56	-0.07	-0.11	-0.17	-0.22	0.2	0.28	-	0.000–0.074
F19	8.25	-0.01	-0.04	-0.07	-0.13	0.11	0.16	-	-0.010–0.195
F20	1.24	-0.07	-0.11	-0.17	-0.27	0.22	0.33	-	-
F21	2.57	0	-0.12	-0.11	-0.16	0.16	0.23	-	-0.012–0.047

Negative values for water table statistics indicate water table level below ground surface, positive values indicate water table is above ground surface. Negative values for vertical hydraulic gradients indicate upward gradients, positive values indicate downward gradients

- Data not available to calculate values

### Modified Flow Accumulation Capacity (MFAC) Outputs

Figure 3 presents MFAC outputs for the four study sites. MFAC classes presented aim to broadly represent relative wetness across each of the study sites, based on relationship between MFAC values and summer D90 levels, as presented in Table 4.

**Table 4:** MFAC classes derived based on predicted summer D90 levels based on regression analysis of summer 2019 data

MFAC Class (km)	Predicted summer D90 based regression analysis of 2019 data illustrated in Fig. 5 (cm below ground level)
0–0.5	≥ 30
0.5–1	25–30
1–2	20–25
2–5	13–20
> 5	< 13

**Fig. 4:**
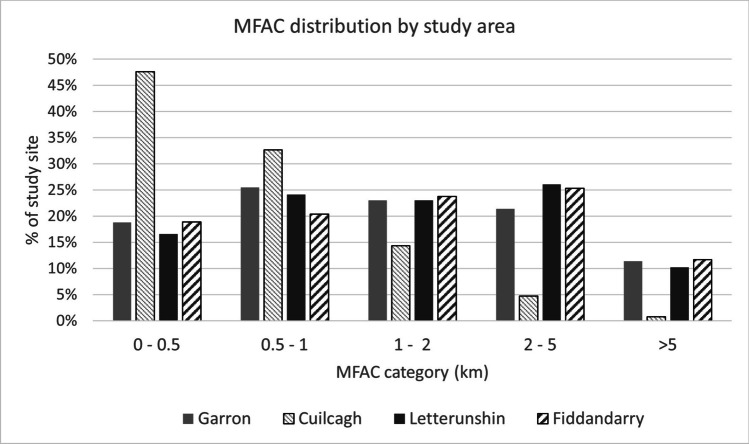
Distribution of Modified Flow Accumulation Capacity (MFAC) categories by percentage area across each of the four study sites

The distribution of the MFAC classes at each site strongly reflects topographic conditions (Fig. 4). Garron is characterised by the presence of a series of relatively flat plateaus having high MFAC values (> 2 km), with the slopes between these flatter areas having lower MFAC values (< 1 km). Letterunshin and Fiddandarry have a comparable distribution of MFAC values, with higher MFAC values (> 2 km) once again associated with the flatter areas, typically characterised by lawns of *Sphagnum spp.*, along with areas of dystrophic pools; lower MFAC values correspond to more steeply sloping areas. Cuilcagh, which has the steepest average slope of the four study sites, had a comparatively smaller proportion of flatter basins, with < 5% of the study catchment being modelled with MFAC values > 2 km.

Summer 2019 D90 levels remained within 20 cm of the ground surface at 21 out of 23 monitoring locations with MFAC values > 1.5 km, while levels were more than 20 cm from the ground surface at 11 out of 13 monitoring locations having MFAC values < 1.5 km. Summer 2019 D90 levels deeper than 25 cm below ground surface were only encountered at locations with a MFAC value of < 0.5 km. A similar trend was found during Summer 2020, albeit with overall summer D90 levels lower below ground level at all monitoring locations due to a more distinct drought period (Table 2), compared to Summer 2019.

Plotting log MFAC against Summer D90 values for individual study areas reveals a roughly linear relationship, with r^2^ values ranging between 0.62 and 0.83. However, due to the limited numbers of sample points at some of the study sites, significant relationships could not be determined at the *P* < 0.05 level for all sites.

**Fig. 5:**
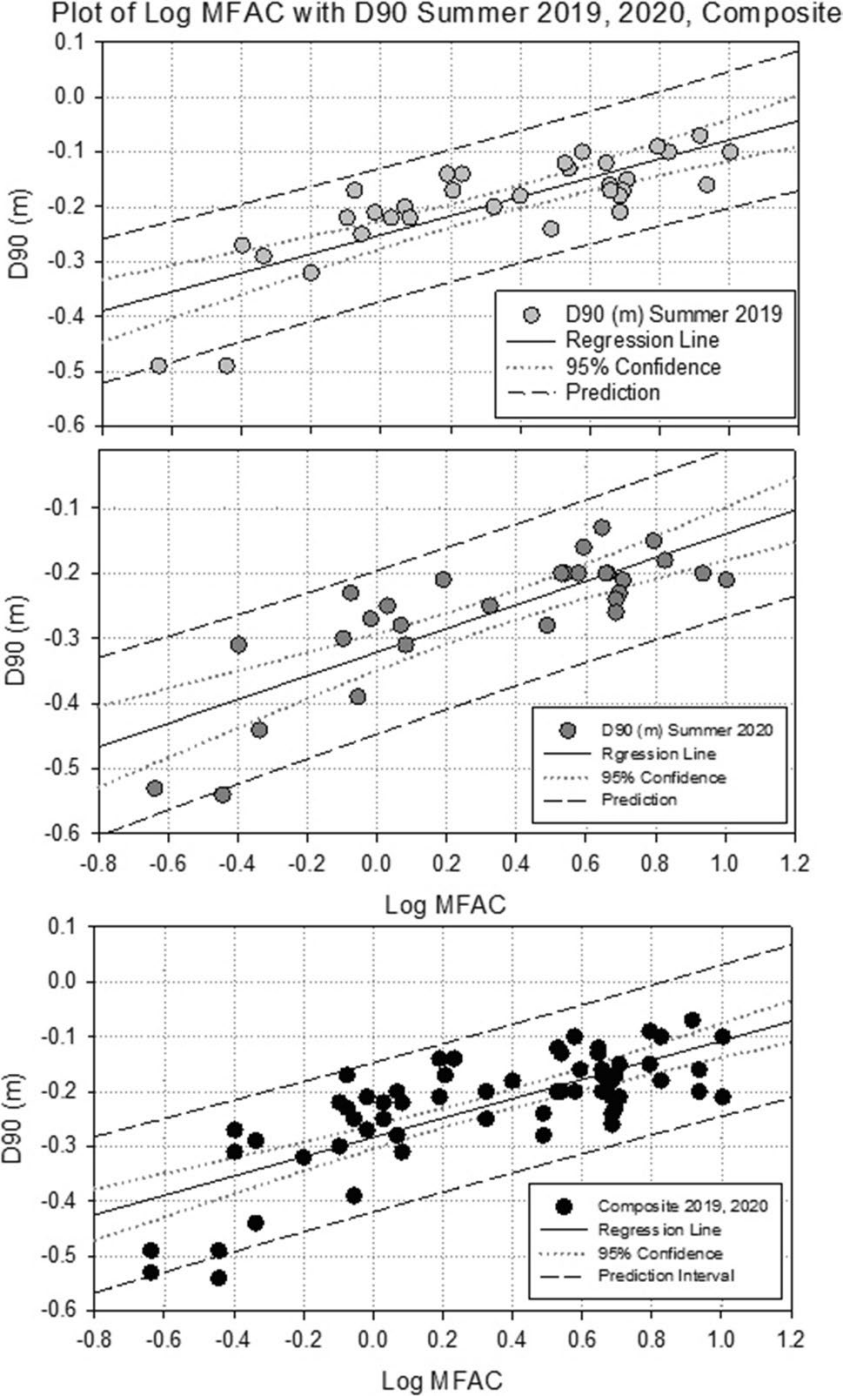
Regression analysis illustrating log Modified Flow Accumulation Capacity (MFAC) outputs with D90 levels for Summer 2019 and Summer 2020. 95% confidence intervals and prediction intervals are illustrated. Results illustrate a trend of deeper D90 levels being associated with low MFAC, values and shallower D90 levels associated with higher MFAC values

Conversely, pooling data from all sites (Fig. 5) revealed a strong significant relationship for summer 2019 (r^2^ = 0.63; F_1,33_ = 55.78, *P* < 0.001) and summer 2020 (r^2^ = 0.66; F_1,31_ = 61.31, *P* < 0.001). Analysis of composite data from both summer 2019 and summer 2020 also revealed a strong significant relationship (r^2^ = 0.57; F_1,66_ = 86.09, *P* < 0.001). The relationship between log MFAC and summer water level fluctuations (D1 minus D99), was not as strong as the relationship with summer D90 levels; however, this was also significant for both summer 2019 (r^2^ = 0.48; F_1,33_ = 30.91, *P* < 0.001) and summer 2020 (r^2^ = 0.55; F_1,31_ = 37.82, *P* < 0.001). Greater water table fluctuations (> 20 cm) were associated with areas with low MFAC values (< 1 km), while areas with the lowest range of fluctuations were associated with high MFAC values. A significant relationship was also identified between summer D90 levels and local surface slope; however, this relationship was not as strong as with log MFAC (summer 2019: r^2^ = 0.51; F_1,33_ = 34.54, *P* < 0.001); summer 2020; (r^2^ = 0.47; F_1,31_ = 27.55, *P* < 0.001).

Comparison of observed summer D90 levels to predicted water tables, based on regression analysis, reveals summer D90 levels to be predicted to within +/-10 cm for 95% of monitoring points for both 2019 and 2020 (Table 5). Despite the significant relationship observed, notable outliers occur, such as F2, F8, F12 and F14, which display anomalously deep water table levels, compared to predicted D90 values using regression analysis.

**Table 5:** Summary of ecological survey results for plots associated with monitoring wells and difference between predicted D90 (based on regression model) and observed summer D90

Plot	MFAC	Predicted summer D90 minus observed summer D90 (2019)	Predicted summer D90 minus observed summer D90 (2020)	Peat thickness (m)	*Sphagnum* spp. cover (%)	*Calluna vulgaris* cover (%)	*Molinia caerulea* cover (%)	Bare peat cover (%)	Depth to H4 (cm)
GA	3.47	-0.02	-0.02	2.93	35	2.5	35	0	15
GB	6.71	0.00	0.01	4.47	12.5	35	0	1	2.5
GC	1.17	-0.03	-0.03	1.6	75	65	0	0	7.5
GD	5.09	0.02	0.02	5.37	22.5	1	92.5	0	2.5
GE	3.79	-0.05	-0.01	2.75	25	25	0	7.5	2.5
GF	1.07	-0.02	-0.06	0.9	7.5	15	92.5	0	2.5
GG	3.92	-	-0.05	3.68	7.5	0	92.5	0	2.5
LA	4.96	0.05	0.04	3.08	12.5	15	1	15	1
LC	0.4	-0.04	-0.08	1.2	12.5	45	45	0	2.5
LD	3.38	-0.03	-0.02	6.65	55	35	2.5	1	22.5
LF	1.21	-0.01	0.01	4.02	25	25	15	1	7.5
LG	4.6	-	0.00	3.01	85	25	1	1	25
CA	0.63	0.04	-	1.7	85	15	1	0	20
CB	1.61	-0.04	-	1.75	85	7.5	1	0	20
CC	1.71	-0.06	-	3.01	45	25	0	2.5	7.5
CD	2.51	0.00	-	2.85	22.5	25	0	2.5	5
F1	10.08	0.03	0.08	3	55	35	25	0	15
F2	0.36	0.17	0.14	1.45	45	15	45	0	10
F3	0.84	-0.09	-0.10	1.7	2.5	25	20	12.5	5
F4	0.46	-0.02	0.06	2.36	25	35	45	1	7.5
F5	6.23	-0.02	-0.02	3.59	25	35	45	1	15
F6	4.54	0.03	0.00	2.8	25	25	65	0	15
F7	4.85	0.05	0.05	3.5	15	45	7.5	7.5	5
F8	0.23	0.13	0.10	1.5	2.5	25	35	2.5	5
F9	0.8	-0.04	-0.03	2.25	25	35	75	0	7.5
F10	2.11	0.01	-0.01	4.36	12.5	25	65	1	7.5
F11	1.55	-0.07	-0.07	2.03	15	15	35	2.5	5
F12	3.08	0.08	0.05	2.42	35	35	65	0	15
F13	0.96	-0.04	-0.05	2.4	1	2.5	95	0	0
F14	4.85	0.08	0.07	2.95	25	45	15	2.5	15
F15	4.43	-0.02	-0.07	3.3	7.5	10	75	0	5
F16	8.63	0.07	0.05	1.99	65	45	45	0	15
F17	0.88	-0.01	0.06	1.95	25	45	45	0	7.5
F18	4.56	0.04	0.02	2.7	45	35	55	0	15
F19	8.25	-0.02	-0.02	3.89	95	12.5	2.5	0	30
F20	1.24	-0.06	-0.03	1.5	25	15	65	0	7.5
F21	2.57	-0.07	-0.08	2.5	85	25	7.5	1	25

- Data not available to calculate values

F2 and F8 have the lowest MFAC values out of all hydrological monitoring plots (< 0.4 km) due to being located on relatively steep slopes (> 10%). Summer 2019 D90 water table levels are ≥ 13 cm deeper than the regression model suggests. In both locations functional (flowing) artificial drainage (contour parallel) occurs within 5 m of the monitoring plot.

Monitoring locations F12 and F14 have high MFAC values (> 3 km), yet summer 2019 D90 levels at both locations are 8 cm deeper than predicted by the regression model. Both monitoring wells occur near functional (flowing) drains. F14 is located 1.4 m from a 0.75 m deep drain, while F12 was located on a slope in an area of higher (contour parallel) drainage density, between a drain 10 m upslope and a second 10 m downslope.

In contrast, summer D90 levels are notably shallower in some areas than the regression model indicates. This includes F3, where the observed summer 2019 D90 level was 9 cm higher than the regression model predicts. F3 has a low MFAC value (0.84 km), occurring on a moderate slope (5.6%), between 0.5 m deep drains running perpendicular to contours.

### Ecological Monitoring Plots

Regression analysis failed to reveal significant relationships between log MFAC values and ecological variables, including *Sphagnum* spp. cover, *Calluna* cover or *Calluna* height (r^2^ values < 0.1, *P* > 0.05). There was also poor relationship between log MFAC and depth to H4 (r^2^ = 0.09); however, this was the only relationship between MFAC and ecological parameters found to be significant (F_1,88_ = 8.87, *P* < 0.01). Furthermore, depth to H4 only exceeded 20 cm at locations where MFAC values were ≥ 1.5 km. At all locations where depth to H4 ≥ 20 cm, *Sphagnum* spp. cover was > 30%, with an average *Sphagnum* spp. cover of 70%. In contrast, although *Sphagnum* spp. cover was up to 75% at some plots where depth to H4 ≤ 5 cm, average *Sphagnum* spp. cover was notably lower at 20%.

For the ecological plots adjacent to hydrological monitoring plots (Table 5), regression analysis revealed poor relationships between summer water table levels (D90, median and D1 minus D99) and *Sphagnum* spp. cover, *Calluna* cover, *Calluna* height and depth to H4 (r^2^ values < 0.1, *P* > 0.05). Depth to H4 exceeded 20 cm at four plots where hydrological monitoring took place (LD, LG, F19 and F20), all of which have shallow and stable water tables (Table 3) and high *Sphagnum* spp. coverage (> 50%).

Results also reveal that despite many locations having a shallow and stable water table, cover of *Sphagnum* spp. remained low at several plots. In contrast, some locations where water table levels are deeper and the range of water table fluctuation is greater, *Sphagnum* spp. cover was found to be notably higher. Examples include plots GB and F15 which had high MFAC values (> 3 km), shallow water tables (summer 2019 median water table level = 6 cm below ground level) and a low range of fluctuations (12–20 cm). Despite these conditions, *Sphagnum* spp. coverage was found to be low at both plots (≤ 12.5%), with high cover (35%) of tall (≥ 35 cm) *Calluna vulgaris* reported at plot GB and high cover (75%) of *Molinia caerulea* reported at plot F15. In the case of F15, this plot is located close of an artificial drain that was found to be heavily infilled with vegetation.

Conversely, plots F2 and CA have low MFAC values (< 0.7 km), deep water tables (summer 2019 median water table level = 15–20 cm below ground level) and larger range of fluctuations (D1 minus D99 ranging from 35 to 57 cm). Despite deep water tables, *Sphagnum* spp. coverage is high (45–85%) at both plots, while cover and height of *Calluna vulgaris* remains low (15% cover and 25 cm height). Furthermore, surveys also identified several plots (e.g., GC and F16) that had a high cover (45–60%) of tall (> 25 cm) *Calluna vulgaris* combined with a high cover (> 60%) of *Sphagnum* spp.

## Discussion

### Relationships Between Modified Flow Accumulation Capacity (MFAC) and Water Table Levels

MFAC modelling reflects the importance of topographic conditions in influencing variability in hydrological regimes across blanket bogs. The relationship between MFAC outputs (Fig. 3) and topographic data (Fig. 2) reveals that areas with high MFAC values correspond to flatter sections of bog and/or areas receiving persistent inputs of water from an upslope contributing catchment; these conditions facilitate greater resilience of water table drawdown during prolonged dry periods, compared to areas with low MFAC values (i.e., steeper areas with more limited upslope contributions).

The ability of MFAC to predict water table levels in different catchments, with differing meteorological conditions, highlights the benefits of the MFAC modelling approach compared to more conventional topographic modelling such as the topographic index. Across all study catchments, MFAC modelling successfully identified areas of consistently elevated (shallow) water tables, compared to those displaying lower (deeper) water table levels and a greater range of fluctuations. Investigations completed by Flynn et al. (2021a) demonstrated that variation in precipitation rates at Garron and Letterunshin due to relief were limited at the catchment scale, suggesting a single catchment-specific climatic correction factor used in the MFAC model was appropriate for each study site.

### Influence of Drainage on Modified Flow Accumulation Capacity (MFAC) Predictions

Despite MFAC proving effective as a predictor of relative surface wetness, some outliers from the observed trends were identified. Most notably, where features were present that lower peatland water tables, such as artificial drains, water table levels proved deeper than anticipated (based on MFAC outputs). Consequently, monitoring points located close to functional drains are not anticipated to reflect water table regimes in comparable topographic settings, where artificial drainage is absent. In other words, it is likely that D90 water table levels at locations where drainage occurred would have been shallower in the absence of drainage, thus leading to an improved relationship with trends generated by other points located away from drains.

However, the hydrological influence of drainage varied, with the results highlighting that where drains are parallel to the contour lines across a slope (such as at F2, F8 and F12), drains are more likely to intercept flow, reducing contributing catchment area, and resulting in deeper water tables. In addition, as highlighted by studies such as Wilson et al. (2010), shallow drains running perpendicular to contours only impact on water table levels within a localised zone of 2–5 m. Consequently, the proximity of monitoring well F14 to the adjacent functional drain (< 1.5 m), is likely to result in lower water tables due to the presence of the artificial drainage.

The regime observed at F14 contrasts with that observed at F3. Plot F3 also occurs on sloping ground, where functional drains are present, yet the monitoring plot is located > 5 m from drains, giving rise to observed water table levels which were notably shallower than anticipated based on MFAC values (and associated regression analysis). Studies such as Holden et al. (2017) and Williamson et al. (2017) further highlighted the limited influence of shallow drains running perpendicular to contours on water tables. Indeed, Williamson et al. (2017) suggested that blanket bogs can display a self-rewetting function, whereby peat subsidence within 4–5 m of drains can result in the peat surface lowering, thus reducing depth to water table. Water table levels at F3 are notably shallower than other locations with similar MFAC values and topographic conditions, suggesting subsidence may have occurred in this area, leading to shallower water table than expected. Despite the shallow water tables observed at plots F3, ecological conditions remain poor, with low cover (2.5%) of *Sphagnum* spp. and a high proportion of bare peat (12.5%). Findings point to localised recovery of hydrological regime, yet a slower recovery of ecological function.

### Deviations Between Modified Flow Accumulation Capacity (MFAC) Predictions and Water Table Levels in Areas Lacking Drainage

Elsewhere, additional monitoring points where the relationship between water table levels and MFAC values prove anomalous, such as at Letterunshin monitoring well LA, cannot be attributed to artificial drainage. This monitoring location occurs on a gentle slope (< 0.6%) and has a high MFAC value (5.0 km), suggesting a persistently shallow water table would be expected. However, summer 2019 D90 levels were 5 cm lower than the regression model predicted. Moreover, a 3.08 m thick sequence of peat underlies this location, suggesting it was likely to be more resilient to prolonged dry periods in the past, to allow a thick sequence of peat to form. Conversely, the anomalously deep water table regime at LA corresponds to ecological metrics generated for the area, which suggest it to be in poor ecological condition. Cover of *Sphagnum spp.* is low (12.5%), while cover of bare peat remains high (15%) and depth to H4 was < 1 cm, indicating an absence of a poorly humified layer of peat at the surface found in more intact locations. Findings suggest anthropogenic disturbance. However, analysis of historical aerial imagery and records of potentially damaging activities during ecological surveys failed to reveal pressures, such as artificial drainage, overgrazing or recent burning.

**Fig. 6:**
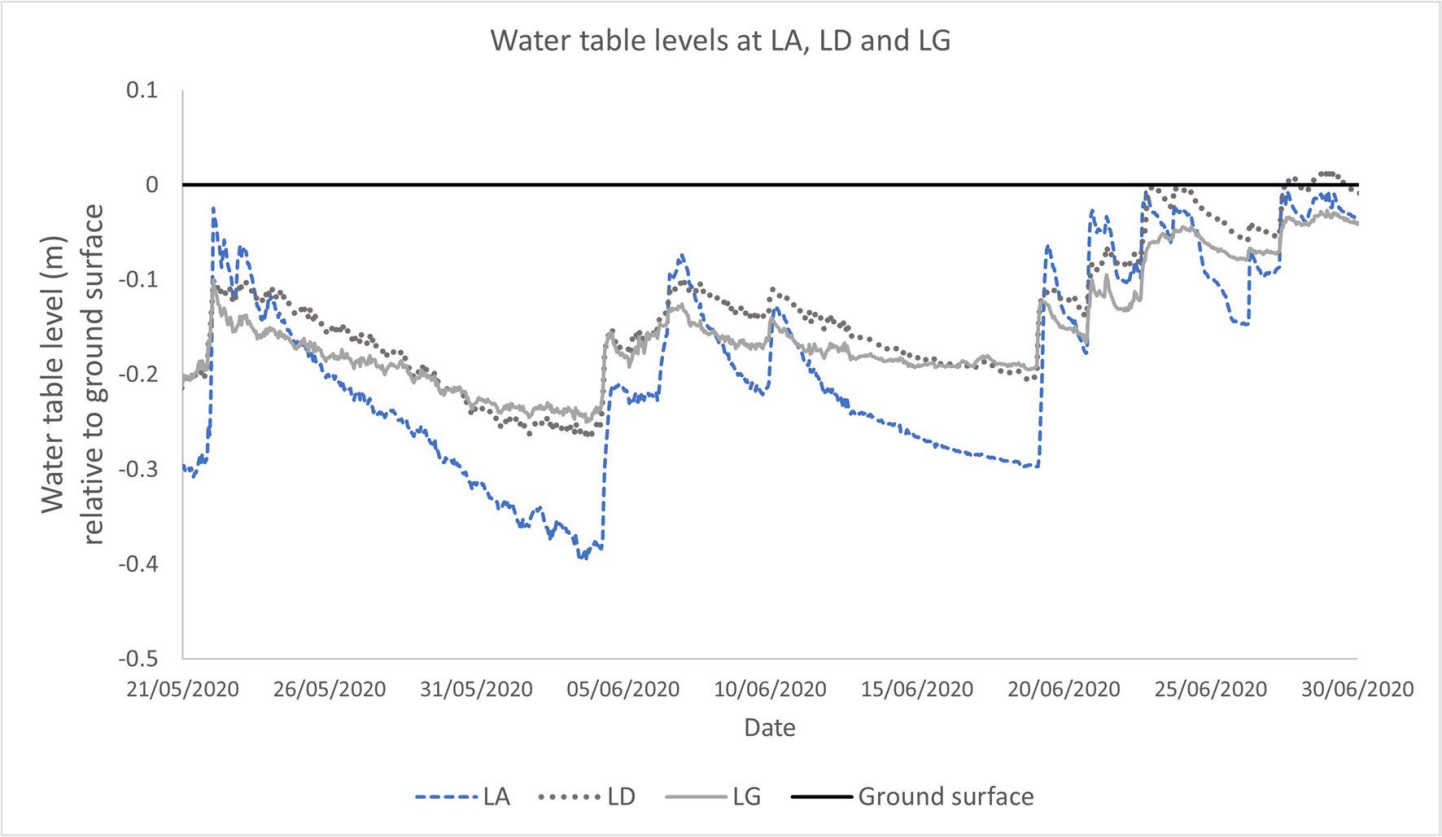
Water table level hydrograph illustrating the difference in rates of decline of water level at monitoring well LA compared to LD and LG despite similar topographic setting and MFAC values

A more detailed review of the dynamics of the water levels at LA revealed that in addition to low summer D90 levels and a large range of water table fluctuations (Table 3), the water table declines more rapidly compared to other locations in a similar topographic setting. For example, when comparing the hydrograph of water table levels at LA to LD and LG, which have similar MFAC values, surface slopes, catchment areas and peat thickness, declines in water table level are more rapid at LA than LD or LG (Fig. 6).

The contrast in hydrograph response, coupled with the poor ecological condition at LA point to another hydrological process, allowing for more rapid groundwater discharge and resulting in deeper water table levels than anticipated. Data collected by Flynn et al. (2022) suggested the peat around LA had a more elevated hydraulic conductivity than that encountered at locations with comparable MFAC values (i.e., LD and LG). Analyses of groundwater monitoring data revealed that horizontal hydraulic gradients at LA, LD and LG proved comparable. Moreover, vertical hydraulic gradients were similar; although slightly more elevated vertical gradients were reported at LA and LD compared to LG. Overall, data indicates that groundwater flowed through peat more rapidly at LA compared to LD or LG. However, more rapid discharge would require continuous connectivity to areas with comparable or more elevated hydraulic conductivity.

Macropores, including peat pipes provide a means of rapidly discharging groundwater from peat that would otherwise reach surface water at a slower rate (Regensburg et al. 2020). Observations, such as those made in the vicinity of LA, thus suggests that a network subsurface preferential flow paths may underlie this part of Letterunshin and affect the water level regime at locations such as LA. The higher hydraulic conductivity of the peat matrix in this area thus provides a means of more rapidly discharging water, than at other locations with comparable MFAC values.

The Mackin et al. (2017b) model and the MFAC model adapted for blanket bogs, assume that losses water loss to depth are negligible. Water quality and hydrogeological measurements, made by Flynn et al. (2021a) at Letterunshin, Cuilcagh and Garron corroborate the assumption that discharge to the substrate underlying the peat forms a minor part (< 5%) of their catchment water balances. However, this assumption fails to consider the presence of macropores, including peat pipes, and the water flowing through them within the peat mass. Where peat (matrix) hydraulic conductivity proves relatively elevated, as at LA (Flynn et al. 2022), the impact of drainage, by more permeable features, such as macropores/peat pipes, on groundwater can extend to greater distances than in less permeable materials (Holden et al. 2006), resulting in lower water table levels.

Data collected at LA highlight a weakness in the MFAC model in being unable to account for deeper subsurface processes, including preferential flow paths which have not expressed themselves in the topography. While larger peat pipes can be identified readily, using topographic data and aerial imagery to identify subsidence and contrasting vegetation, detection of smaller branched networks of preferential flow paths through small pipes and macropores poses a greater challenge. Regensburg et al. (2020) found that topography alone provided a poor indication of the catchment area for areas impacted by piping and that a branched network of peat pipes, not visible based on topography, can occur across a wide area. Where these networks cannot be identified, they cannot be incorporated into the MFAC model. This limits the utility of MFAC in areas affected by interconnected macropore development. On the other hand, it highlights the potential for MFAC to be a useful tool in hydrological characterisation of blanket bogs, by indicating differences between anticipated surface wetness and observations on the ground.

Overall, despite the deviations observed in areas with artificial drains and macropores, results support the hypothesis that areas with a higher MFAC values have shallower, more stable, water tables. Results of regression analysis, revealing a globally significant relationship between MFAC and water table levels, and groundwater fluctuations, across a range of blanket bogs. This proves particularly valid in areas lacking anthropogenic hydrological disturbances, such as artificial drainage. Critically, a stronger relationship was found between water table levels and MFAC values, than using slope alone, highlighting the value of considering climate and contributing catchment along with surface slope.

### Relationships Between Modified Flow Accumulation Capacity (MFAC) and Ecological Parameters

Despite the significant relationship observed between MFAC and water table observations, the relationship between MFAC and ecological parameters proved poor. However, it is important to acknowledge that a poor relationship was also found between observed water table levels and ecological parameters. This is believed to reflect both legacy anthropogenic disturbance and limitations of the vegetation survey methodology employed. The latter was adapted using key parameters employed in the assessment of Irish raised bogs (Fernandez et al. 2014) to enable rapid assessment of ecological conditions.

On Irish raised bogs Swenson et al. (2019) noted *Sphagnum* spp. cover strongly correlated with whether peat is accumulating, and therefore if carbon is being sequestered (Fernandez et al. 2014; Regan et al. 2020). However, ecological parameters employed in the current study were broad (e.g., total *Sphagnum spp.* cover rather than reporting cover for individual species which occur in differing hydrological niches). Consequently, it is apparent that this approach has resulted in over-simplification, whereby species that are not likely to be peat-accumulating were not distinguished from species more likely to be associated with peat formation. This is particularly evident from plots such as GC and F16, which have a high cover of *Sphagnum* spp. and tall *Calluna vulgaris*, suggesting that *Sphagnum* spp. are more likely to be species adapted to drier conditions.

On the other hand, evidence from ecological monitoring suggests that despite selecting relatively intact blanket bogs for this study, some areas continue experience the legacy of ongoing effects from historically detrimental activities including drainage, burning and overgrazing. These past activities resulted in areas with suitable hydrological conditions for *Sphagnum spp.*, having a high cover of bare peat or high cover of species indicative of drier conditions (e.g., tall *Calluna vulgaris* or widespread M*olinia caerulea)*. As highlighted by plots GB and F15, areas with high MFAC values and shallow water tables were not always associated with high *Sphagnum* spp. cover. In both cases, low *Sphagnum* spp. coverage, combined with high cover of species indicating drier conditions, points to slow recovery from past anthropogenic pressures. It is likely that in both cases water tables would have been lower in the past, thus permitting tall *Calluna vulgaris* to develop at GB and M*olinia caerulea* to become dominant at F15. In the case of F15, the evidence of drainage, infilled with vegetation, suggests drainage initially lowered water table levels, permitting M*olinia caerulea* to become dominant. As the drain infilled with vegetation, the drainage function has reduced, leading to improved hydrological conditions, yet a slow recovery of *Sphagnum* spp. due to the dominance of M*olinia caerulea.*

Despite the poor relationship observed, an important relationship between MFAC, water table levels and depth to H4 was found. Greatest depth to H4 occurred in areas with high MFAC values and shallow, stable water tables. These areas are believed to reflect areas of least anthropogenic disturbance in the past, meaning a thick layer of poorly humified peat remained close to the surface. In contrast, locations with high MFAC values, shallow water tables and shallow depth to H4, are more likely to reflect past anthropogenic disturbance.

## Conclusions

Adaptation of the MFAC model to blanket bogs has demonstrated the important relationship between topographic conditions and water table levels. More specifically, MFAC has proven effective in predicting areas that are more likely to have shallower and more stable summer water levels. The success of the MFAC modelling approach, adopted to a range of sites following corrections for climatic variability and surface water drainage, points to its wider potential for application to predict water table regimes in blanket bogs elsewhere. This includes across the network of blanket bogs in Ireland and in settings supporting comparable climatic conditions.

Given the importance of shallow, stable water tables, MFAC demonstrates potential to assist peatland managers in identifying high priority areas for restoration, where increases in water table levels and associated peat accumulating vegetation may be more easily re-established. To optimise use of resources, areas with high MFAC values display greater potential to have these conditions and should therefore be prioritised for restoration over areas with lower MFAC values.

While there were deviations between MFAC predictions of surface wetness and observed water table levels, particularly where features, such as artificial drainage, lower water tables, MFAC predictions can provide peatland managers with useful insights into hydrological processes that are not immediately obvious from remote sensing or ground surveys. This includes identification of legacy effects of anthropogenic disturbance, resulting in anomalously wet areas lacking peat accumulating vegetation, and areas having elevated MFAC values, yet unable to support shallow and stable water table regimes. The latter scenario points to the occurrence of subsurface hydrological processes, unaccounted for in the MFAC model (e.g., discharge via unidentified macropores/peat pipes). The capacity to identify these processes thus assists in the targeting of site-specific investigations.

Considerable scope remains to evaluate the effectiveness of the adapted MFAC model, including the impacts of historic anthropogenic activity. Acquisition of further topographic/water table data from other sites promises to further evaluate the confidence in which the model may be applied more widely. However, data acquisition should be appropriately focused to ensure optimal use of resources.

Poor relationships between MFAC and ecological parameters reflects the need for further characterisation of ecological conditions that accurately reflect peat-forming conditions in blanket bog landscapes. Paleo-ecological studies could provide further insight into the potential influence past impacting activities may have had on these areas, particularly in areas with low *Sphagnum* cover, despite high MFAC values combined with shallow and stable water tables.

### Supplementary Information

Below is the link to the electronic supplementary material.

ESM 1 (DOCX 6.58 MB)

## Data Availability

The datasets generated during the study are available from the corresponding author on reasonable request.

## References

[CR1] Acreman M, Holden J (2013). How wetlands affect floods. Wetlands.

[CR2] Allen RG, Pereira LS, Raes D, Smith M (1998). FAO irrigation and drainage paper no. 56.

[CR3] Allott T, Auñón J, Dunn C, Evans M, Labadz J, Lunt P, MacDonald M, Nisbet T, Owen R, Pilkington M, Proctor S, Shuttleworth E, Walker J (2019) Peatland catchments and natural flood management. Report to the IUCN UK Peatland Programme’s Commission of Inquiry on Peatlands Update. Available at: https://research.bangor.ac.uk/portal/files/27930383/Allott_et_al_2019_IUCN_COI_Peatlands_and_NFM_FULL_REPORT.pdf. Accessed 22/02/2022

[CR4] Allott TEH, Evans MG, Lindsay JB, Agnew CT, Freer JE, Jones A, Parnell M (2009) Water tables in Peak District blanket peatlands. Moors for the Future Report No 17. Available at: https://www.escholar.manchester.ac.uk/api/datastream?publicationPid=uk-ac-man-scw:18867&datastreamId=FULL-TEXT.PDF. Accessed 02/09/2023

[CR5] Armstrong A, Holden J, Kay P, Foulger M, Gledhill S, McDonald AT, Walker A (2009). Drain-blocking techniques on blanket peat: a framework for best practice. Journal of Environmental Management.

[CR6] Bain CG, Bonn A, Stoneman R, Chapman S, Coupar A, Evans M, Gearey B, Howat M, Joosten H, Keenleyside C, Labadz J, Lindsay R, Littlewood N, Lunt P, Miller CJ, Moxey A, Orr H, Reed M, Smith P, Swales V, Thompson DBA, Thompson PS, Van de Noort R, Wilson JD, Worrall F (2011) IUCN UK Commission of Inquiry on Peatlands. IUCN UK Peatland Programme, Edinburgh. Available at: https://repository.uel.ac.uk/download/ef97e4b6cc318de731500e7c8c62292f1bd017412670e8fe10e89a2aea2a6714/2498467/IUCN%20UK%20Commission%20of%20Inquiry%20on%20Peatlands%20Full%20Report%20spv%20web.pdf. Accessed 22/02/2022

[CR7] Beven KJ, Kirkby MJ (1979). A physically based, variable contributing area model of basin hydrology. Hydrological Sciences Bulletin.

[CR8] Charman D (2002). Peatlands and environmental change.

[CR9] Chow AT, Tanji KK, Gao S (2003). Production of dissolved organic carbon (DOC) and trihalomethane (THM) precursor from peat soils. Water Research.

[CR10] Creevy AL, Payne RJ, Andersen R, Rowson JG (2020) Annual gaseous carbon budgets of forest-to-bog restoration sites are strongly determined by vegetation composition. Sci Total Environ 705:35863. 10.1016/j.scitotenv.2019.135863 10.1016/j.scitotenv.2019.135863 31972925

[CR11] Crowley W, Smith GF, Mackin F, Regan S, Fernandez-Valverde F, Eakin M (2021). Recovery of the vegetation of a cutover raised bog in Ireland following rewetting measures. Biology and Environment: Proceedings of the Royal Irish Academy.

[CR12] Cushnan H (2018) Quantifying the baseline conditions and restoration potential of Irish raised bogs through hydrogeological and geophysical methods. Unpublished PhD thesis. School of Natural and Built Environment, The Queen’s University of Belfast

[CR13] Douglas C, Garvey L, Kelly L, O’Sullivan A (1989). A survey to locate blanket bogs of scientific interest in County Kerry and County Sligo.

[CR14] ESRI (2022) An overview of the Map Algebra toolset. Available at: https://desktop.arcgis.com/en/arcmap/latest/tools/spatial-analyst-toolbox/an-overview-of-the-map-algebra-toolset.htm. Accessed 02/03/2022

[CR15] EU (2013) Interpretation Manual of European Union Habitats. EUR 28, European Commission, DG Environment, Brussels. Available at: https://ec.europa.eu/environment/nature/legislation/habitatsdirective/docs/Int_Manual_EU28.pdf. Accessed 02/03/2022

[CR16] Evans CD, Peacock M, Baird AJ, Artz R, Brown E, Burden A, Callaghan N, Chapman PJ, Cooper HM, Coyle M, Cumming A, Dixon S, Helfter C, Heppell C, Holden J, Gauci V, Grayson RP, Jones D, Kaduk J, Levy P, Matthews R, McNamara N, Misselbrook T, Oakley S, Page S, Rayment M, Ridley LM, Stanley K, Williamson J, Worrall F, Morrison R (2021). Overriding water table control on managed peatland greenhouse gas emissions. Nature.

[CR17] Evans M, Warburton J (2011) Geomorphology of upland peat: Erosion, form and landscape change. Wiley. https://www.wiley.com/en-us/Geomorphology+of+Upland+Peat%3A+Erosion%2C+Form+and+Landscape+Change-p-9781444391695

[CR18] Fernandez F, Connolly K, Crowley W, Denyer J, Duff K, Smith G (2014) Raised bog monitoring and assessment survey 2013. Irish Wildlife Manuals, No. 81. National Parks and Wildlife Service, Department of Arts, Heritage and Gaeltacht, Dublin, Ireland. ISSN 1393–6670. Available at: https://www.npws.ie/sites/default/files/publications/pdf/IWM81_0.pdf. Accessed 14/02/2022

[CR19] Flynn R, Mackin F, McVeigh C, Renou-Wilson F (2022). Impacts of a mature forestry Plantation on Blanket Peatland Runoff Regime and Water Quality. Hydrological Processes.

[CR20] Flynn R, McVeigh C, Mackin F, Renou-Wilson F (2021). Sources of Stream Base flow in blanket peat covered catchments. Journal of Hydrology.

[CR21] Flynn R, Mackin F, Renou-Wilson F (2021). Towards the Quantification of Blanket Bog Ecosystem Services to Water. EPA Research Report No. 378.

[CR22] Gallego-Sala AV, Prentice IC (2013). Blanket peat biome endangered by climate change. Nature Climate Change.

[CR23] Graniero PA, Price JS (1999). Distribution of bog and heath in a Newfoundland blanket bog complex: topographic limits on the hydrological processes governing blanket bog development. Hydrology and Earth System Science.

[CR24] Greenlee DD (1987). Raster and Vector Processing for Scanned Linework. Photogrammetric Engineering Remote and Sensing.

[CR25] Hammond RF (1981) The peatlands of Ireland. An Foras Taluntais. Available at: https://www.teagasc.ie/media/website/environment/soil/Peatlands-of-Ireland.pdf. Accessed 10/02/2022

[CR26] Holden J, Evans MG, Burt TP, Horton M (2006). Impact of land drainage on peatland hydrology. Journal of Environmental Quality.

[CR27] Holden J, Shotbolt L, Bonn A, Burt TP, Chapman PJ, Dougill AJ, Fraser EDG, Hubacek K, Irvine B, Kirkby MJ, Reed MS, Prell C, Stagl S, Stringer LC, Turner A, Worrall F (2007). Environmental change in moorland landscapes. Earth-Sci Reviews.

[CR28] Holden J, Walker J, Evans MG, Worrall F, Bonn A (2008) A compendium of peat restoration and management projects. A report to the Department for Environment, Food & Rural Affairs (DEFRA), London. Report No. SP0556. Available at: https://www.iucn-uk-peatlandprogramme.org/sites/default/files/Defra%20report_0.pdf. Accessed 29 Dec 2023

[CR29] Holden J, Wallage ZE, Lane SN, McDonald AT (2011). Water table dynamics in undisturbed, drained and restored blanket peat. Journal of Hydrology.

[CR30] Holden J, Green SM, Baird AJ, Grayson RP, Dooling GP, Chapman PJ, Evans CD, Peacock M, Swindles G (2017). The impact of ditch blocking on the hydrological functioning of blanket peatlands. Hydrol Process.

[CR31] Ingram HAP, Gore AJP (1983). Hydrology. Ecosystems of the World 4A, mires: swamp, bog, Fen and moor.

[CR32] Joint Nature Conservation Committee (2022) Garron Plateau SAC. Available at: https://sac.jncc.gov.uk/site/UK0016606. Accessed: 10/02/2022

[CR33] Joint Nature Conservation Committee (2011) Towards an assessment of the state of UK Peatlands. JNCC report No. 445. Available at: https://data.jncc.gov.uk/data/f944af76-ec1b-4c7f-9f62-e47f68cb1050/JNCC-Report-445-FINAL-WEB.pdf. Accessed 29 Dec 2023

[CR34] Kelly ML (1993) Hydrology, Hydrochemistry and Vegetation of Two Raised Bogs in Co Offaly. Ph.D. Thesis. Trinity College, Dublin

[CR35] Kritzler UH, Artz R, Johnson D (2016). Soil CO2 efflux in a degraded raised bog is regulated by water table depth rather than recent plant assimilate. Mires and Peat.

[CR36] Kuemmerlen M, Moorkens EA, Piggott JJ (2022). Assessing remote sensing as a tool to monitor hydrological stress in Irish catchments with Freshwater Pearl Mussel populations. Science of Total Environment.

[CR37] Lane SN, Brookes CJ, Kirkby MJ, Holden J (2004). A network-index-based version of TOPMODEL for use with high resolution digital topographic data. Hydrological Processes.

[CR38] Lindsay R (1995) Bogs: the ecology, classification and conservation of ombrotrophic mires. Scottish Natural Heritage, Edinburgh, UK

[CR39] Mackin F, Barr A, Rath P, Eakin M, Ryan J, Jeffrey R, Fernandez Valverde F (2017a) Best practice in raised bog restoration in Ireland. Irish Wildlife Manuals, No. 99, National Parks and Wildlife Service, Department of Culture, Heritage and the Gaeltacht, Ireland (2017) Available at: https://www.npws.ie/sites/default/files/publications/pdf/IWM99_RB_Restoration_BestPracticeGuidance.pdf. Accessed: 10/02/2022

[CR40] Mackin F, Flynn R, Barr A, Fernandez Valverde F (2017). Use of geographical information system-based hydrological modelling for development of a raised bog conservation and restoration programme. Ecological Engineering.

[CR41] McKeown R, Corbett P (2017) Garron Plateau SAC UK0016606. Conservation Objectives. Northern Ireland Environment Agency (NIEA). Available at: https://www.daera-ni.gov.uk/sites/default/files/publications/doe/Conservation%20Objectives%20%282017%29.%20%20Garron%20Plateau%20SAC.%20%20Version%202.1%20-%20amendment%2012.10.2017.%20PDF.PDF. Accessed: 10/02/2022

[CR42] NI Water (2019). Co-operation Across Borders for Biodiversity: restoration of the blanket bog in Dungonnell catchment.

[CR43] NPWS (2016) Ox mountains bogs SAC: site synopsis. National Parks and Wildlife Service, Dublin

[CR44] Nugent KA, Strachan IB, Strack M, Roulet NT, Rochefort L (2018). Multi-year net ecosystem carbon balance of a restored peatland reveals a return to carbon sink. Global Change Biology.

[CR45] O’Driscoll C, Sheahan J, Renou-Wilson F, Croot P, Pilla F, Misstear B, Xiao L (2018). National scale assessment of total trihalomethanes in Irish drinking water. Journal of Environmental Management.

[CR46] Oosterwoud M, van der Ploeg M, van der Schaaf S, van der Zee S (2017). Variation in hydrologic connectivity as a result of microtopography explained by discharge to catchment size relationship. Hydrological Processes.

[CR47] Parry LE, Holden J, Chapman PJ (2014). Restoration of blanket peatlands. Journal of Environmental Management.

[CR48] Perrin P, Roche J, Barron S, Daly O, Hodd R, Muldoon C, Leyden K (2013a) National survey of upland habitats (Phase 3, 2012–2013). Site Report No. 13: Cuilcagh – Anierin Uplands cSAC (000584), Cos. Cavan and Leitrim. National Parks and Wildlife Service, Dublin

[CR49] Perrin P, Roche J, Barron S, Daly O, Hodd R, Muldoon C, Leyden K (2013b) National survey of upland habitats (Phase 3, 2012–2013). Site Report No. 10: Ox Mountains Bogs cSAC (002006), Cos. Mayo and Sligo. National Parks and Wildlife Service, Dublin

[CR50] Regan S, Flynn R, Gill L, Naughton O, Johnston P (2019). Impacts of groundwater drainage on peatland subsidence and its ecological implications on an Atlantic raised bog. Water Resources Research.

[CR51] Regan S, Swenson M, O’Connor M, Gill L (2020) Ecohydrology, greenhouse gas dynamics and restoration guidelines for degraded raised bogs. EPA Research Report No. 342. Environmental Protection Agency, Wexford. Available at: https://www.epa.ie/publications/research/biodiversity/Research_Report_342.pdf. Accessed 12/02/2022

[CR52] Regensburg TH, Chapman PJ, Pilkington MG, Chandler DM, Evans MG, Holden J (2020). Effects of pipe outlet blocking on hydrological functioning in a degraded blanket peatland. Hydrol Process.

[CR53] Renou-Wilson F, Bolger T, Bullock C, Convery F, Curry J, Ward S, Wilson D, Müller C (2011) BOGLAND: Sustainable Management of Peatlands in Ireland STRIVE Report. Environmental Protection Agency, Wexford. Available at: https://www.epa.ie/publications/research/land-use-soils-and-transport/STRIVE_75_web_SC.pdf. Accessed: 22/02/2022

[CR54] Roulet NT, Lafleur PM, Richard PJH, Moore T, Humphreys ER, Bubier J (2007). Contemporary carbon balance and late Holocene carbon accumulation in a northern peatland. Global Change Biology.

[CR55] Schouten MCG (2002). Conservation and restoration of raised bogs, Geological, Hydrological and Ecological studies.

[CR56] Sibson R, Barnett V (1981). A brief description of natural neighbor interpolation. Interpolating Multivariate Data.

[CR57] Sottocornola M, Kiely G (2010). Hydro-meteorological controls on the CO2 exchange variation in an Irish blanket bog. Agricultural and Forest Meteorology.

[CR58] Swenson MM, Regan S, Bremmers DTH, Lawless J, Saunders M, Gill LW (2019). Carbon balance of a restored and cutover raised bog: implications for restoration and comparison to global trends. Biogeosciences.

[CR59] van der Schaaf S, Schouten MGC (2002). Bog hydrology. Conservation and restoration of raised bogs. Geological hydrological and ecological studies.

[CR60] van der Schaaf S, Streefkerk J (2002) Relationships between biotic and abiotic conditions. In: Schouten MCG (ed) Conservation and restoration of raised bogs, Geological, Hydrological and Ecological Studies. Department of the Environment and Local Government/Staatsbosbeheer, Dublin

[CR61] von Post L (1922). Sveriges Geologiska Undersöknings torvinventering och några av dess hitills vunna resultat. Svenska Mosskulturföreningens Tidskrift.

[CR62] Walsh S (2012) A Summary of climate averages 1981–2010 for Ireland, Climatological Note No.14, Met Éireann, Dublin. Available at: https://www.met.ie/climate/30-year-averages. Accessed: 10/02/2022

[CR63] Williamson J, Rowe E, Reed D, Ruffino L, Jones P, Dolan R, Buckingham H, Norris D, Astbury S, Evans C (2017). Historical peat loss explains limited short-term response of drained blanket bogs to rewetting. Journal of Environmental Management.

[CR64] Wilson D, Dixon SD, Artz RRE, Smith TEL, Evans CD, Owen HJF, Archer E, Renou-Wilson F (2015). Derivation of greenhouse gas emission factors for peatlands managed for extraction in the Republic of Ireland and the United Kingdom. Biogeosciences.

[CR65] Wilson L, Holden J, Johnstone I, Armstrong A, Morris M (2011). The impact of drain blocking on an upland blanket bog during Storm and drought events, and the importance of sampling-scale. Journal of Hydrology.

[CR66] Wilson L, Wilson J, Holden J, Johnstone I, Armstrong A, Morris M (2010). Recovery of water tables in Welsh blanket bog after drain blocking: discharge rates, time scales and the influence of local conditions. Journal of Hydrology.

[CR67] Xu J, Morris PJ, Liu J, Holden J (2018). Hotspots of peatland-derived potable water use identified by global analysis. Nature Sustainability.

