# Supplementary material for: Application of a GIS-Based Hydrological Model to Predict Surface Wetness of Blanket Bogs
Source: Wetlands (Wilmington). 2024 Jan 3;44(1):10. doi: 10.1007/s13157-023-01765-5 (PMC10764537; doi:10.1007/s13157-023-01765-5)
Supplement: Supplementary file 1 — (DOCX 6.58 MB) [file 13157_2023_1765_MOESM1_ESM.docx]

**Supplementary material**

**
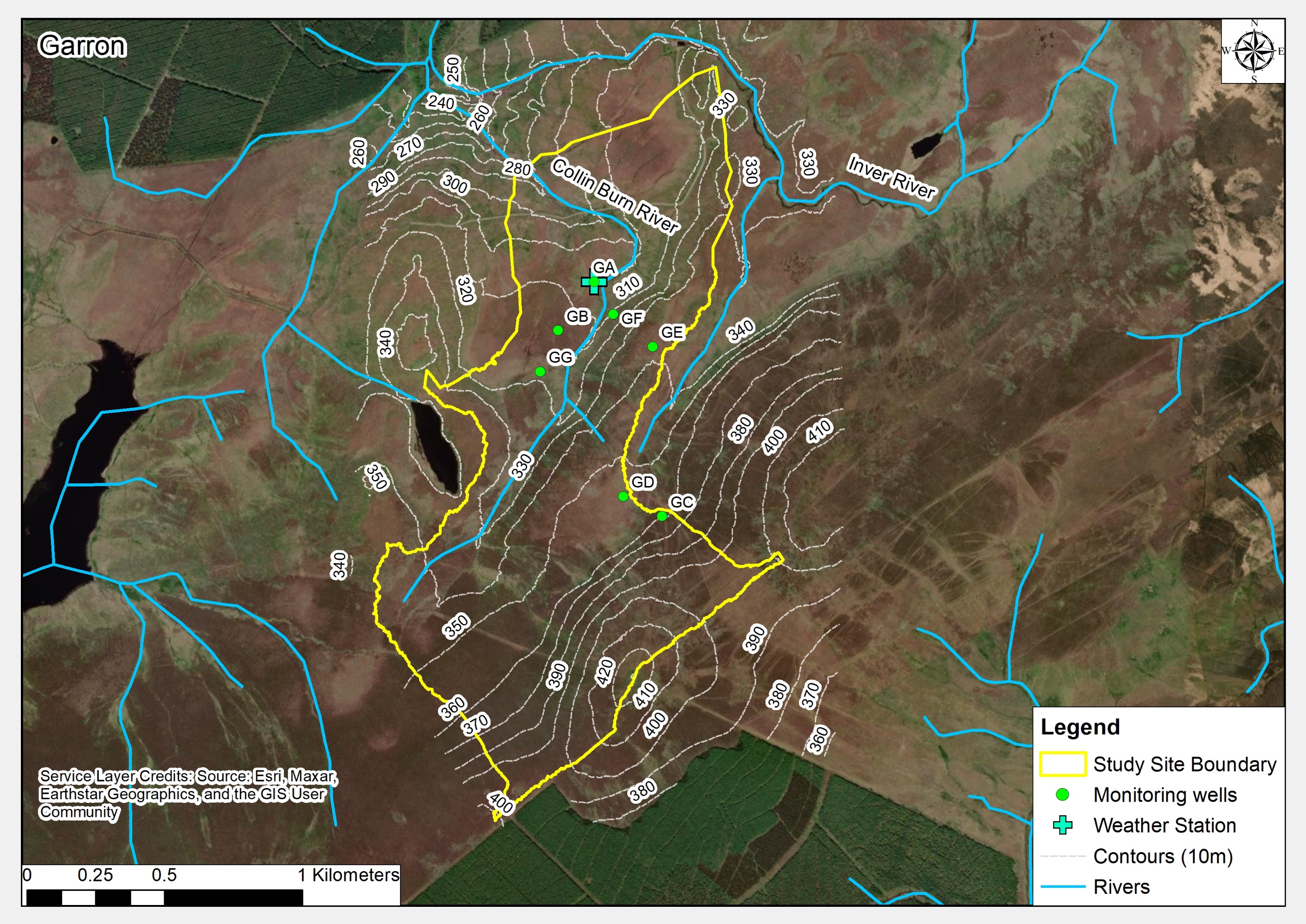
**

**Figure S1 Detailed site overview map for Garron**

**
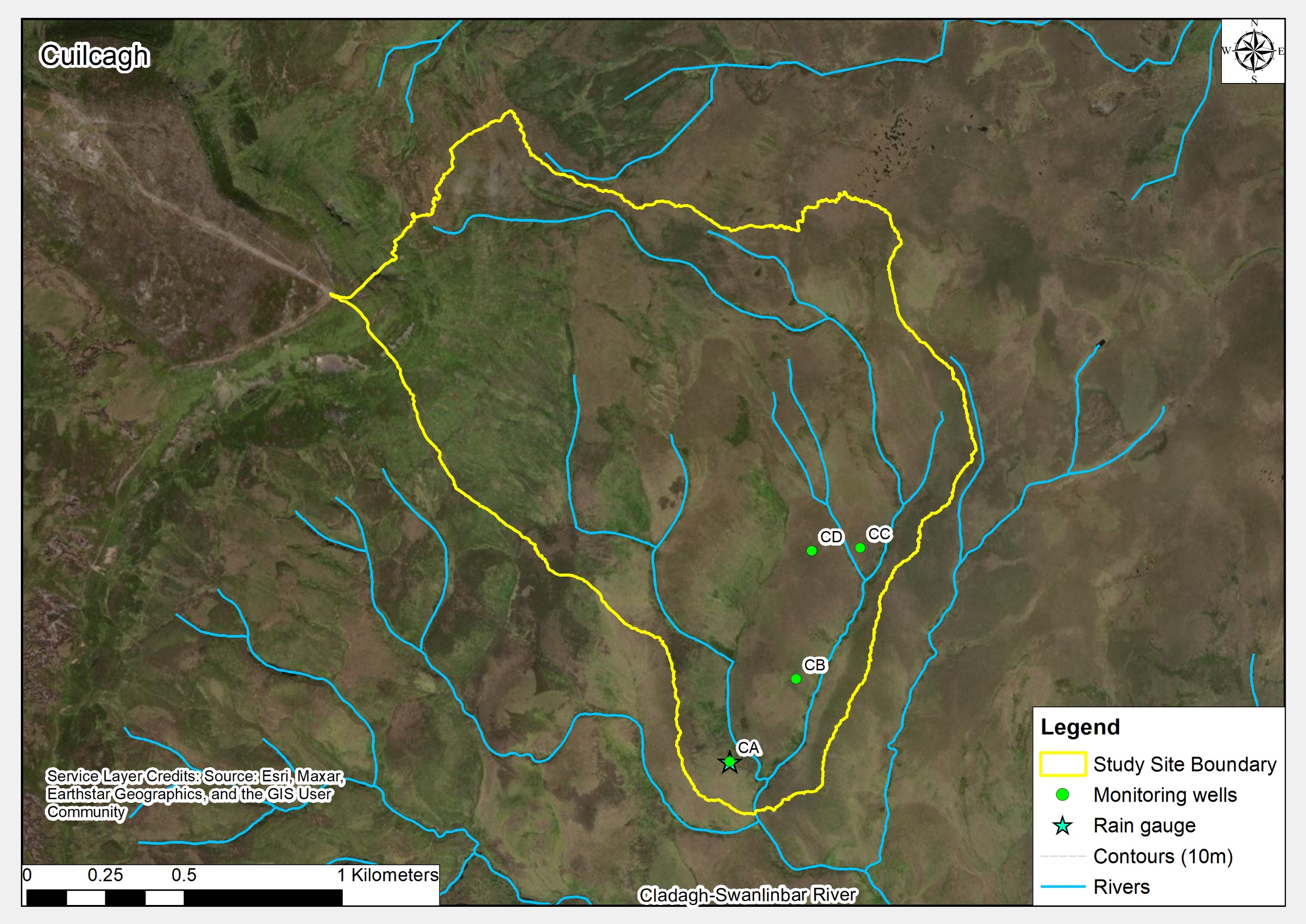
**

**Figure S2 Detailed site overview map for Cuilcagh**

**
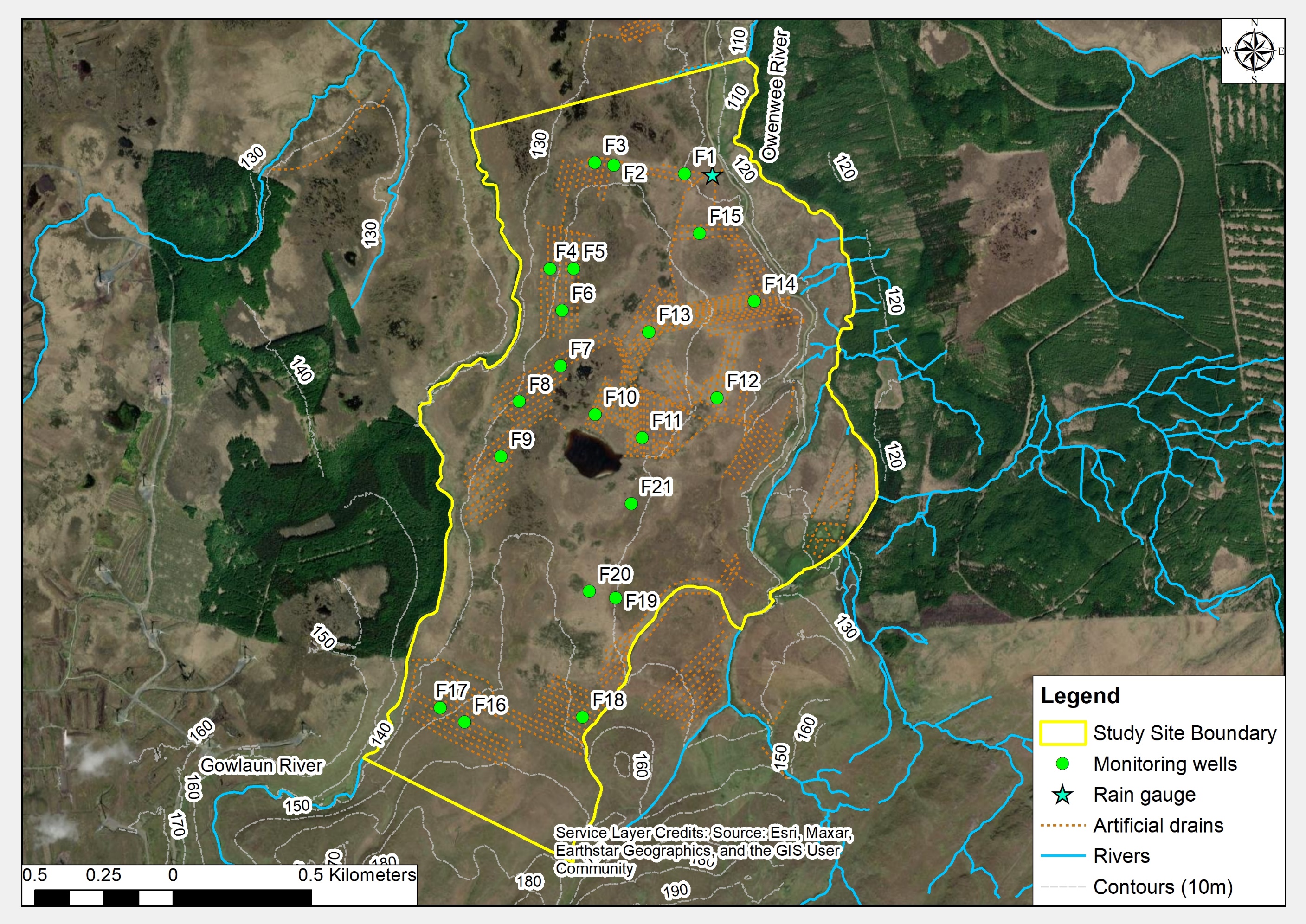
**

**Figure S3 Detailed site overview map for Fiddandarry**

**
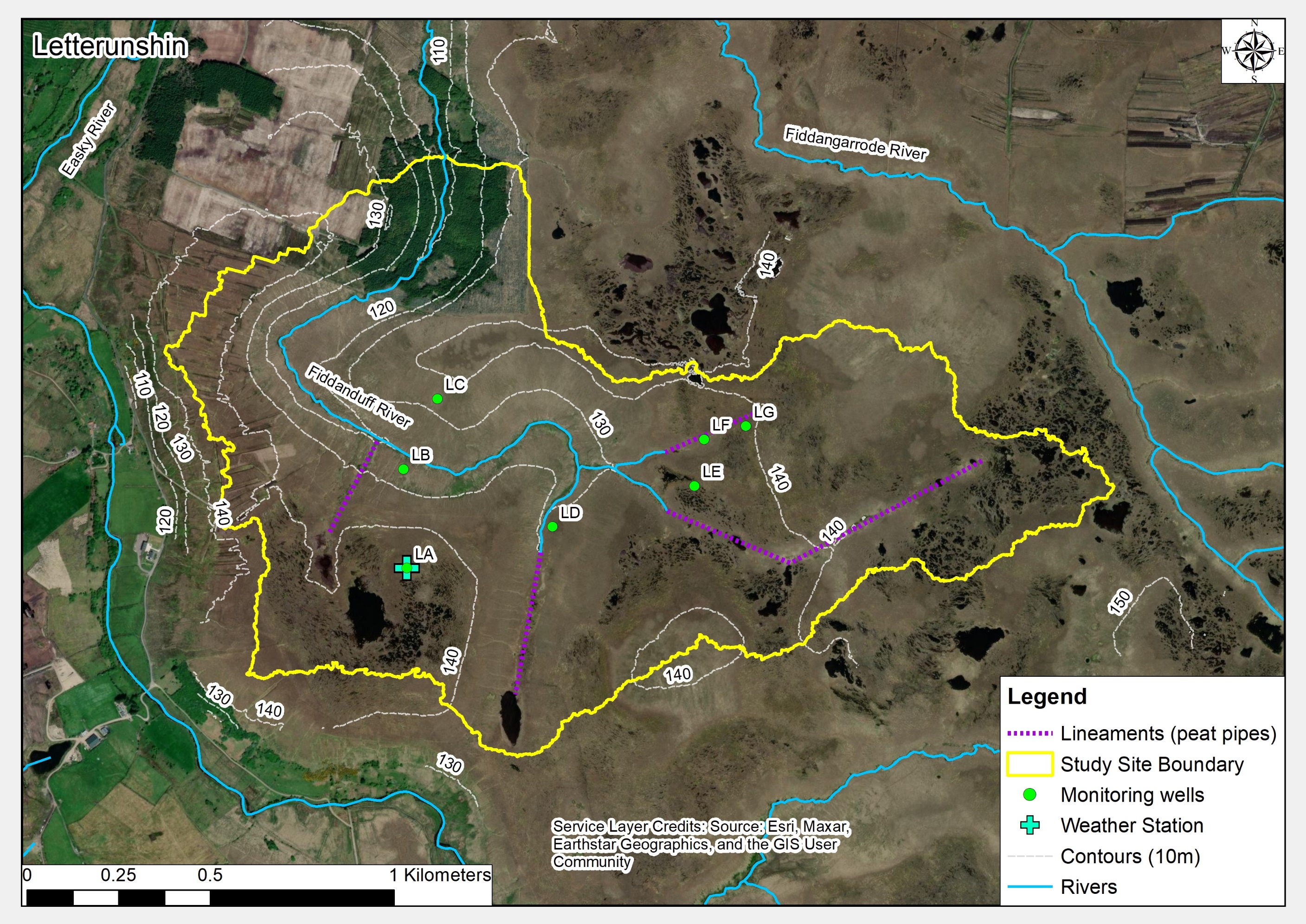
**

**Figure S4 Detailed site overview map for Letterunshin**

**Table S1 Monthly meteorological measurements**

| **Garron** | **Year** | **Jan** | **Feb** | **Mar** | **Apr** | **May** | **Jun** | **Jul** | **Aug** | **Sep** | **Oct** | **Nov** | **Dec** | **Total** |
| --- | --- | --- | --- | --- | --- | --- | --- | --- | --- | --- | --- | --- | --- | --- |
| Precipitation (mm) | 2019 | 86.8 | 99.7 | 157.0 | 87.6 | 58.0 | 88.0 | 87.6 | 124.2 | 119.2 | 99.0 | 162.2 | 98.8 | 1268.1 |
| PE (mm) |  | 8.7 | 19.5 | 34.0 | 58.4 | 76.9 | 79.9 | 74.6 | 63.5 | 40.3 | 25.6 | 12.3 | 8.5 | 502.4 |
| Rain days (≥1 mm) |  | 18 | 12 | 20 | 10 | 13 | 16 | 13 | 20 | 21 | 18 | 17 | 18 | 196 |
| Wet days (≥5 mm) |  | 5 | 8 | 12 | 8 | 6 | 8 | 6 | 11 | 12 | 4 | 11 | 8 | 99 |
| Precipitation (mm) | 2020 | 64.2 | 176.0 | 84.2 | 27.6 | 30.8 | 113.4 | 108.2 | 168.4 | 96.6 | 209.6 | 110.8 | 112.8 | 1302.6 |
| PE (mm) |  | 9.1 | 18.1 | 31.7 | 64.0 | 95.5 | 75.0 | 65.9 | 58.5 | 46.2 | 22.4 | 9.9 | 7.3 | 503.6 |
| Rain days (≥1 mm) |  | 13 | 25 | 16 | 7 | 8 | 15 | 18 | 16 | 16 | 22 | 21 | 19 | 196 |
| Wet days (≥5 mm) |  | 5 | 12 | 7 | 3 | 2 | 8 | 8 | 11 | 7 | 17 | 10 | 9 | 99 |
|  |  |  |  |  |  |  |  |  |  |  |  |  |  |  |
| **Letterunshin** | **Year** | **Jan** | **Feb** | **Mar** | **Apr** | **May** | **Jun** | **Jul** | **Aug** | **Sep** | **Oct** | **Nov** | **Dec** | **Total** |
| Precipitation (mm) | 2019 | 100.6 | 102.4 | 186.8 | 107.3 | 68.3 | 113.5 | 101.4 | 209.3 | 119.3 | 138.3 | 129.3 | 150.8 | 1526.9 |
| PE (mm) |  | 10.6 | - | 37.6 | 83.3 | 96.0 | 92.6 | 72.3 | 70.3 | 57.2 | 39.6 | 20.8 | 18.7 | 599.1 |
| Rain days (≥1 mm) |  | 19 | 16 | 19 | 13 | 14 | 15 | 17 | 25 | 23 | 22 | 20 | 21 | 224 |
| Wet days (≥5 mm) |  | 9 | 7 | 17 | 9 | 5 | 9 | 7 | 14 | 10 | 11 | 11 | 9 | 118 |
| Precipitation (mm) | 2020 | 119.8 | 284.3 | 118.0 | 25.0 | 34.8 | 147.0 | 101.5 | 124.5 | 78.5 | 252.0 | 159.0 | 226.5 | 1670.7 |
| PE (mm) |  | 22.4 | 30.7 | 51.1 | 93.3 | 133.0 | 78.6 | 76.4 | 76.9 | 62.2 | 24.0 | 3.0 | 22.1 | 673.7 |
| Rain days (≥1 mm) |  | 19 | 27 | 18 | 9 | 6 | 16 | 22 | 19 | 17 | 24 | 21 | 27 | 225 |
| Wet days (≥5 mm) |  | 10 | 21 | 8 | 1 | 2 | 9 | 10 | 5 | 8 | 15 | 11 | 18 | 118 |
|  |  |  |  |  |  |  |  |  |  |  |  |  |  |  |
| **Fiddandarry*** | **Year** | **Jan** | **Feb** | **Mar** | **Apr** | **May** | **Jun** | **Jul** | **Aug** | **Sep** | **Oct** | **Nov** | **Dec** | **Total** |
| Precipitation (mm) | 2019 | 99.2 | 101.0 | 182.4 | 105.7 | 68.1 | 111.7 | 104.2 | 215 | 114.2 | 133.4 | 128.4 | 139.2 | 1502.6 |
| Rain days (≥1 mm) |  | 18 | 15 | 18 | 12 | 13 | 14 | 16 | 24 | 22 | 21 | 17 | 21 | 212 |
| Wet days (≥5 mm) |  | 9 | 7 | 15 | 9 | 5 | 9 | 6 | 13 | 10 | 11 | 12 | 9 | 114 |
| Precipitation (mm) | 2020 | 111 | 265 | 114.4 | 19.6 | 30.8 | 159.6 | 110 | 123 | 80.4 | 245.2 | 155.6 | 220.6 | 1635.2 |
| Rain days (≥1 mm) |  | 17 | 26 | 19 | 4 | 8 | 16 | 21 | 19 | 18 | 23 | 20 | 26 | 217 |
| Wet days (≥5 mm) |  | 9 | 17 | 10 | 1 | 2 | 9 | 10 | 5 | 6 | 14 | 10 | 16 | 109 |
|  |  |  |  |  |  |  |  |  |  |  |  |  |  |  |
| **Cuilcagh** | **Year** | **Jan** | **Feb** | **Mar** | **Apr** | **May** | **Jun** | **Jul** | **Aug** | **Sep** | **Oct** | **Nov** | **Dec** | **Total** |
| Precipitation (mm) | 2019 | 141 | 200.8 | 332.2 | 115.6 | 17.8 | 74.6 | 105.4 | 316.6 | 245.2 | 228 | 182.6 | 275.2 | 2235.0 |
| Rain days (≥1 mm) |  | 21 | 18 | 23 | 14 | 6 | 13 | 15 | 21 | 23 | 21 | 20 | 21 | 216 |
| Wet days (≥5 mm) |  | 10 | 12 | 19 | 8 | 0 | 5 | 7 | 18 | 17 | 11 | 13 | 15 | 135 |

* Note records at Fiddandarry supplemented by establishing the relationship between records at Letterunshin and Fiddandarry using regression analysis (r2 > 0.91).

**Figure S5 Water level duration curves for summer and winter monitoring periods for all monitoring plots illustrating water levels as a function of the proportion of time a particular water level (relative to ground surface) is equalled or exceeded (Negative values indicate depths below ground surface, positive values indicate height above ground surface measured from a fixed point).**


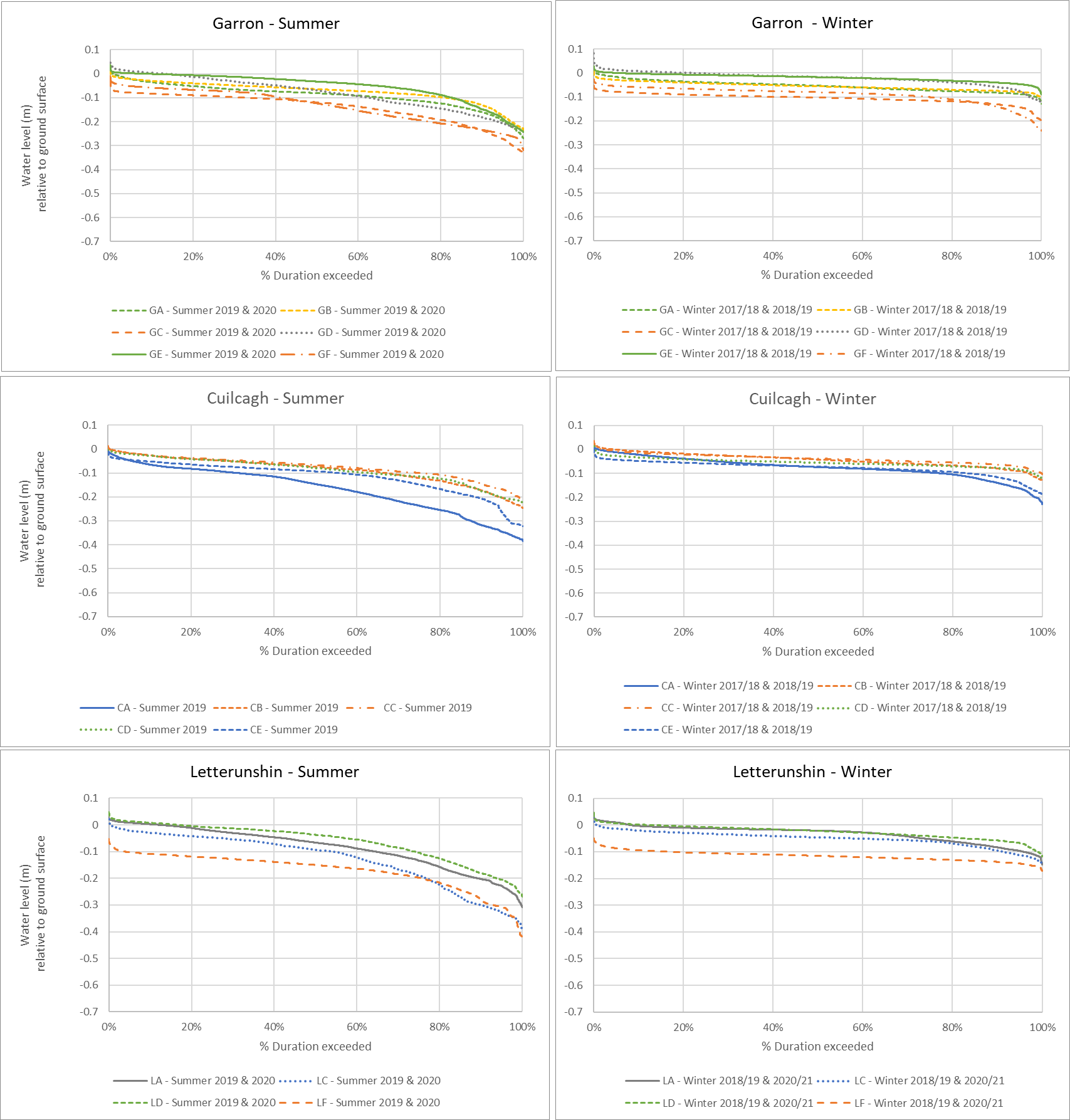

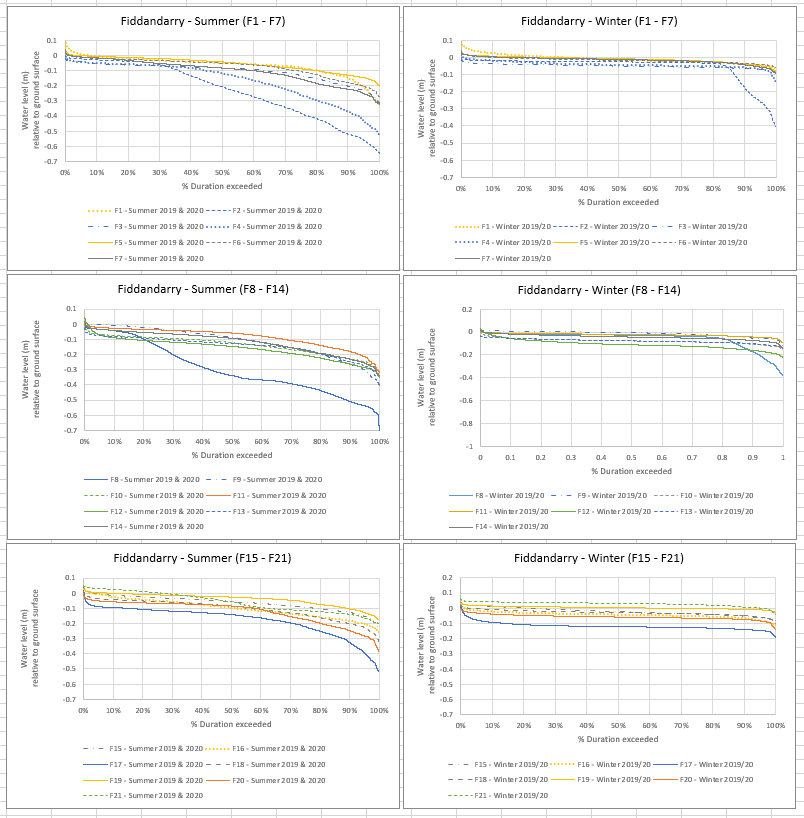


**Table S2 Horizontal hydraulic gradients recorded at Garron, Cuilcagh and Letterunshin (only reported for sites with monitoring wells installed in triplicate arrangement)**

| **Nest** | **03/07/2019** | **06/07/2019** | **29/08/2019** | **20/11/2019** | **26/01/2020** | **18/03/2020** | **09/04/2020** | **20/05/2020** | **13/06/2020** | **15/07/2020** | **30/08/2020** | **10/04/2020** | **22/12/2020** |
| --- | --- | --- | --- | --- | --- | --- | --- | --- | --- | --- | --- | --- | --- |
| **GA** | 0.028 | 0.029 | 0.029 | 0.032 | 0.030 | 0.029 | 0.054 | 0.031 | 0.029 | 0.030 | 0.051 | 0.029 | 0.031 |
| **GB** | 0.028 | 0.027 | 0.029 | 0.029 | 0.029 | 0.029 | 0.027 | 0.029 | 0.028 | 0.027 | 0.028 | 0.029 | 0.028 |
| **GC** | 0.169 | 0.167 | 0.168 | 0.167 | 0.169 | 0.167 | 0.168 | 0.176 | 0.168 | 0.167 | 0.171 | 0.158 | 0.168 |
| **GD** | 0.018 | 0.015 | 0.018 | 0.016 | 0.018 | 0.016 | 0.015 | 0.016 | 0.016 | 0.018 | 0.015 | 0.017 | 0.015 |
| **GE** | 0.021 | 0.012 | 0.026 | 0.014 | 0.016 | 0.015 | 0.017 | 0.018 | 0.018 | 0.016 | 0.015 | 0.016 | 0.018 |
| **GF** | 0.129 | 0.130 | 0.134 | 0.128 | 0.133 | 0.131 | 0.131 | 0.138 | 0.130 | 0.129 | 0.142 | 0.126 | 0.129 |
| **GG** | 0.024 | 0.027 | 0.030 | 0.060 | 0.026 | 0.026 | 0.025 | 0.026 | 0.022 | 0.022 | 0.025 | 0.025 | 0.025 |
|  |  |  |  |  |  |  |  |  |  |  |  |  |  |
|  | **06/02/2019** | **16/05/2019** | **14/07/2019** | **06/09/2020** | **02/12/2020** | **29/03/2021** | **21/05/2021** | **02/01/2022** |  |  |  |  |  |
| **LA** | 0.017 | 0.013 | 0.008 | 0.013 | 0.011 | 0.016 | 0.013 | 0.016 |  |  |  |  |  |
| **LC** | 0.09 | 0.096 | 0.096 | 0.095 | 0.096 | 0.095 | 0.095 | 0.096 |  |  |  |  |  |
| **LD** | 0.012 | 0.019 | 0.019 | 0.012 | 0.013 | 0.013 | 0.013 | 0.012 |  |  |  |  |  |
| **LF** | 0.041 | 0.041 | 0.041 | 0.042 | 0.041 | 0.039 | 0.042 | 0.043 |  |  |  |  |  |
| **LG** | 0.029 | 0.019 | 0.018 | 0.018 | 0.019 | 0.02 | 0.02 | 0.019 |  |  |  |  |  |
|  |  |  |  |  |  |  |  |  |  |  |  |  |  |
|  | **03/07/2019** | **06/07/2019** | **29/08/2019** |  |  |  |  |  |  |  |  |  |  |
| **CA** | 0.132 | 0.119 | 0.089 |  |  |  |  |  |  |  |  |  |  |
| **CB** | 0.031 | 0.029 | 0.058 |  |  |  |  |  |  |  |  |  |  |
| **CC** | 0.045 | 0.056 | 0.061 |  |  |  |  |  |  |  |  |  |  |
| **CD** | 0.028 | 0.027 | 0.022 |  |  |  |  |  |  |  |  |  |  |

**Table S3 Vertical hydraulic gradients recorded at Garron, Letterunshin and Fiddandarry (only reported for nest with a shallow monitoring well and piezometer)**

|  | **03/07/2019** | **06/07/2019** | **29/08/2019** | **20/11/2019** | **26/01/2020** | **18/03/2020** | **09/04/2020** | **20/05/2020** | **13/06/2020** | **15/07/2020** | **30/08/2020** | **10/04/2020** | **22/12/2020** |
| --- | --- | --- | --- | --- | --- | --- | --- | --- | --- | --- | --- | --- | --- |
| **GA** | 0.355 | 0.438 | 0.368 | 0.405 | 0.328 | 0.355 | 0.407 | 0.380 | 0.414 | 0.362 | 0.310 | 0.358 | 0.342 |
| **GB** | 0.002 | 0.234 | 0.774 | 0.412 | 0.406 | 0.657 | 0.755 | 0.691 | 0.667 | 0.641 | 0.604 | 0.628 | 0.596 |
| **GD** | 0.075 | 0.171 | 0.076 | 0.085 | 0.080 | 0.078 | 0.066 | 0.072 | 0.109 | 0.103 | 0.086 | 0.097 | 0.073 |
| **GE** | 0.065 | 0.368 | 0.019 | 0.032 | 0.079 | 0.080 | 0.075 | 0.083 | 0.104 | 0.097 | 0.070 | 0.072 | 0.072 |
| **GG** | 0.097 | 0.313 | 0.109 | -0.010 | 0.025 | 0.109 | 0.123 | 0.115 | 0.122 | 0.129 | 0.123 | 0.124 | 0.103 |
|  |  |  |  |  |  |  |  |  |  |  |  |  |  |
|  | **06/02/2019** | **16/05/2019** | **14/07/2019** | **06/09/2020** | **02/12/2020** | **29/03/2021** | **21/05/2021** |  |  |  |  |  |  |
| **LA** | 0.074 | -0.010 | 0.263 | 0.038 | 0.024 | 0.050 | 0.064 |  |  |  |  |  |  |
| **LD** | 0.079 | 0.050 | 0.153 | 0.062 | 0.055 | 0.067 | 0.145 |  |  |  |  |  |  |
| **LF** | 0.052 | 0.048 | 0.153 | 0.111 | 0.093 | 0.089 | 0.116 |  |  |  |  |  |  |
| **LG** | 0.000 | -0.005 | 0.027 | 0.044 | 0.028 | 0.025 | 0.039 |  |  |  |  |  |  |
|  |  |  |  |  |  |  |  |  |  |  |  |  |  |
|  | **10/10/2019** | **27/01/2020** | **12/06/2020** | **09/09/2020** | **16/02/2021** | **16/03/2021** |  |  |  |  |  |  |  |
| **F1** | 0.319 | 0.273 | 0.342 | 0.308 | 0.427 | 0.404 |  |  |  |  |  |  |  |
| **F5** | 0.005 | 0.047 | 0.000 | 0.010 | 0.000 | 0.005 |  |  |  |  |  |  |  |
| **F7** | 0.025 | 0.186 | 0.105 | 0.025 | 0.040 | 0.000 |  |  |  |  |  |  |  |
| **F10** | 0.040 | 0.034 | 0.006 | 0.020 | 0.043 | 0.030 |  |  |  |  |  |  |  |
| **F12** | 0.040 | 0.033 | 0.033 | 0.047 | 0.187 | 0.053 |  |  |  |  |  |  |  |
| **F14** | 0.000 | 0.067 | 0.056 | 0.000 | 0.067 | 0.010 |  |  |  |  |  |  |  |
| **F15** | 0.073 | 0.094 | 0.105 | 0.026 | 0.058 | 0.016 |  |  |  |  |  |  |  |
| **F18** | 0.022 | 0.000 | 0.000 | 0.016 | 0.074 | 0.038 |  |  |  |  |  |  |  |
| **F19** | 0.195 | 0.000 | 0.000 | -0.010 | 0.058 | 0.017 |  |  |  |  |  |  |  |
| **F21** | 0.000 | 0.000 | 0.047 | 0.023 | 0.006 | -0.012 |  |  |  |  |  |  |  |

**Table S4 Complete results from ecological surveys of monitoring plots. Ranges of parameters were recorded with mid-values used for regression analysis**

|  | **Plot ID** | **Microtopography** | **Depth to H4 (cm)** | **Peat thickness (m)** | **Ground softness** | **Total cover *Molinia* (%)** | **Total cover other grasses (%)** | **Total cover *Calluna* (%)** | **Height of *Calluna* (cm)** | **Total cover *Erica* spp. (%)** | **Height of *Erica* spp. (cm)** | **Total cover of *Sphagna* (%)** | **Total cover of bare peat (%)** | **Total cover of peat with algal mat (%)** | **Evidence of land use** | **MFAC (km)** |
| --- | --- | --- | --- | --- | --- | --- | --- | --- | --- | --- | --- | --- | --- | --- | --- | --- |
| Cuilcagh | **C_1** | Low hummocks and *Sphagnum* lawns | 20 | 1.55 | 3 (soft) | <1 | 40-50 | 10-20 | 20-30 | 5-10 | 5-10 | 80-90 | 0 | <1 | Light grazing, *Juncus squarrosus* (5%), sheep dropping in plot | 0.4 |
| Cuilcagh | **C_2** | Low hummocks | 0-5 | 0.45 | 2 (hard to soft) | 0-5 | 15-30 | 10-20 | 20-30 | 5-10 | 5-10 | 30-40 | 0-5 | 0 | 35 | 0.1 |
| Cuilcagh | **C_3** | Low hummocks and hollows | 10-15 | >3.0 | 3 (soft) | 0 | 0 | 0-5 | 20-30 | 0-5 | 5-10 | 40-50 | 0-5 | <1 | Light grazing | 1.9 |
| Cuilcagh | **C_4** | Low hummocks and *Sphagnum* lawns | >30 | 2.7 | 3 (soft) | <1 | 30-40 | 0-5 | 20-30 | 0-5 | 5-10 | 80-90 | 0 | 0-5 | Light grazing | 1.7 |
| Cuilcagh | **C_5** | Low hummocks | 5 | 2.15 | 2 (hard to soft) | 0 | 40-50 | 15-25 | 20-30 | 10-20 | 5-10 | 10-20 | 5-10 | 0 | Light grazing | 0.4 |
| Cuilcagh | **C_6** | Low hummocks | 5-10 | 1.2 | 2 (hard to soft) | 0-5 | 25-35 | 30-40 | 30-40 | 0-5 | 5-10 | 30-40 | 0 | 0-5 | Light grazing | 0.1 |
| Cuilcagh | **C_7** | Low hummocks | 5 | 0.45 | 2 (hard to soft) | 10-20 | 0 | 5-10 | 10-20 | 0-5 | 5-10 | 70-80 | 0-5 | 0 | Moderate grazing | 0.1 |
| Cuilcagh | **C_8** | Low hummocks | 5 | 2.4 | 2 (hard to soft) | 0 | 20-30 | 20-30 | 20-30 | 5-10 | 5-10 | 20-25 | 0-5 | 0-5 | Light grazing | 1.0 |
| Cuilcagh | **C_11** | Low hummocks | 5-10 | 3 | 3 (soft) | 0 | 15-25 | 20-30 | 10-20 | 0-5 | 5-10 | 40-45 | 0-5 | <1 | Light grazing | 1.2 |
| Cuilcagh | **C_12** | Low hummocks and *Sphagnum* lawns | 20-25 | 2.7 | 3 (soft) | 10-20 | 15-25 | 0-5 | 20-30 | 0-5 | 5-10 | 70-80 | 0-5 | <1 | Light grazing, sheep droppings | 1.8 |
| Cuilcagh | **C_13** | Low hummocks | 10-15 | 1.6 | 2 (hard to soft) | 0-5 | 5-15 | 20-30 | 20-30 | 0-5 | 5-10 | 80-90 | 0 | 0 | Light grazing, *Juncus squarrosus* (5-10), sheep dropping in plot | 0.3 |
| Cuilcagh | **C_14** | Low hummocks | 10-15 | >2.55 | 3 (soft) | <1 | 15-25 | 10-20 | 20-30 | 5-10 | 5-10 | 80-90 | 0-5 | <1 | Light grazing | 1.2 |
| Cuilcagh | **C_15** | Low hummocks | 5 | 2.15 | 2 (hard to soft) | <1 | 25-35 | 20-30 | 20-30 | 0-5 | 5-10 | 25-30 | <1 | <1 | Light grazing | 0.4 |
| Cuilcagh | **C_17** | Low hummocks | 20 | >3.0 | 3 (soft) | <1 | 45-55 | 5-10 | 20-30 | 0-5 | 5-10 | 80-90 | 0 | 0 | None | 0.9 |
| Cuilcagh | **C_18** | Low hummocks | 5-10 | 0.45 | 2 (hard to soft) | 10-20 | 25-35 | 10-20 | 20-30 | 5-10 | 5-10 | 50-60 | <1 | 0 | Light grazing, *Juncus effusus* (5-10), sheep dropping in plot | 0.2 |
| Cuilcagh | **C_2A** | Low hummocks | 0-5 | 1.45 | 1 (hard) | 0 | 40-50 | 60-70 | 30-40 | 0-5 | 20-30 | 5-10 | 0 | <1 | Light grazing | 0.7 |
| Cuilcagh | **C_1A** | Low hummocks and hollows | 0.10-0.15 | >3.0 | 3 (soft) | <1 | 5-15 | 15-20 | 20-30 | 0-5 | 5-10 | 80-90 | <1 | <1 | None | 0.7 |
| Cuilcagh | **C_2B** | Low hummocks | 0-5 | 1.65 | 2 (hard to soft) | <1 | n/a | <1 | 20-30 | 0-5 | 5-10 | 30-40 | <1 | n/a | Light grazing. *Juncus squarrosus* (5%). Likely to indicate overgrazing (perhaps more in the past) | 0.1 |
| Cuilcagh | **C_2C** | Low hummocks and *Sphagnum* lawns | 10-20 | 2.2 | 3 (soft) | 10-20 | n/a | 0 | 0 | 0-5 | 5-10 | 80-90 | 0 | n/a | Light grazing. Sheep droppings in square. | 0.3 |
| Fiddandarry | F1 | Low hummocks/ *Sphagnum* hollows within an active flush system | 15 | 3 | 3 (soft) | 20-30 | 25-35 | 30-40 | 30-40 | 1 | 10-20 | 50-60 | 0 | 0 | Shallow infilling drain (15m) | 9.8 |
| Fiddandarry | F2 | Low hummocks/*Narthecium* flats | 10 | 1.45 | 2 (hard to soft) | 40-50 | 25-35 | 10-20 | 20-30 | 5-10 | 10-20 | 40-50 | 0 | 0 | Shallow infilling drain (10m) | 0.4 |
| Fiddandarry | F3 | Low hummocks/*Narthecium* flats | <5 | 1.7 | 1 (hard) | 20 | 40-50 | 20-30 | 10-20 | 1 | 10-20 | 0-5 | 10-15 | <1 | Shallow (0.75cm) drain (5m) | 0.9 |
| Fiddandarry | F4 | Low hummocks/*Narthecium* flats | 5-10 | 2.36 | 1 (hard) | 40-50 | 15-25 | 30-40 | 10-20 | 0-5 | 10-20 | 20-30 | 1 | <1 | Shallow (0.75cm) drain (5m) | 0.5 |
| Fiddandarry | F5 | High & Low hummocks/ *Sphagnum & open* water pools | 10-20 | 3.59 | 3 (soft) | 40-50 | 15-25 | 30-40 | 5-10 | 1 | 5-10 | 20-30 | 1 | <1 | Shallow infilling drain (5m) | 5.1 |
| Fiddandarry | F6 | Low hummocks/*Narthecium* hollows | 10-20 | 2.8 | 2 (hard to soft) | 60-70 | 0 | 20-30 | 20-30 | 0-5 | 20-30 | 20-30 | 0 | 0 | Shallow infilling drain (10m) | 4.5 |
| Fiddandarry | F7 | Low hummocks/open water pools/*Narthecium* hollows | <5 | 3.5 | 2 (hard to soft) | 5-10 | 30-40 | 40-50 | 5-10 | 5-10 | 5-10 | 10-20 | 5-10 | 0-5 | Shallow (0.75cm) drain (5m) | 49.8 |
| Fiddandarry | F8 | Low hummocks/*Narthecium* flats | <5 | 1.5 | 1 (hard) | 30-40 | 5-15 | 20-30 | 10-20 | 0-5 | 10-20 | 0-5 | 0-5 | <1 | Shallow (0.75cm) drain (10m) | 0.2 |
| Fiddandarry | F9 | High hummocks/*Molinia* hollows | 5-10 | 2.25 | 2 (hard to soft) | 70-80 | 0 | 30-40 | 10-20 | 0-5 | 10-20 | 20-30 | 0 | 0 | Shallow (0.75cm) drain (10m) | 0.8 |
| Fiddandarry | F10 | High hummocks/*Narthecium* hollows | 5-10 | 4.36 | 2 (hard to soft) | 60-70 | 0 | 20-30 | 10-20 | 0-5 | 10-20 | 10-15 | 1 | <1 | Shallow (0.75cm) drain (5m) | 2.3 |
| Fiddandarry | F11 | Low hummocks/*Narthecium* flats | <5 | 2.03 | 1 (hard) | 30-40 | 15-25 | 10-20 | 10-20 | 0-5 | 10-20 | 10-20 | 0-5 | <1 | Shallow (0.75cm) drain (5m) | 1.0 |
| Fiddandarry | F12 | High hummocks/*Molinia* hollows | 10-20 | 2.42 | 2 (hard to soft) | 60-70 | 0 | 30-40 | 40-50 | 0-5 | 10-20 | 30-40 | 0 | 0 | Shallow infilling drain (5m) | 3.0 |
| Fiddandarry | F13 | *Molinia* tussocks | 0 | 2.4 | 2 (hard to soft) | 90-100 | 0 | 0-5 | 20-30 | <1 | 10-20 | <1 | 0 | 0 | Shallow (0.75cm) drain (5m) | 1.2 |
| Fiddandarry | F14 | Low hummocks & *Narthecium* hollows | 10-20 | 2.95 | 3 (soft) | 10-20 | 25-35 | 40-50 | 10-20 | 0-5 | 10-20 | 20-30 | 0-5 | <1 | Shallow (0.75cm) drain (1m), small amount of trampling | 5.2 |
| Fiddandarry | F15 | High hummocks/*Molinia* hollows | <5 | 3.3 | 2 (hard to soft) | 70-80 | 0 | 10 | 20-30 | <1 | 10-20 | 5-10 | 0 | 0 | Shallow infilling drain (15m) | 5.6 |
| Fiddandarry | F16 | High hummocks/*Molinia* & *Sphagnum* hollows | 10-20 | 1.99 | 2 (hard to soft) | 40-50 | 5-15 | 40-50 | 20-30 | 0-5 | 10-20 | 60-70 | 0 | 0 | Shallow infilling drain (10m) | 8.9 |
| Fiddandarry | F17 | High hummocks/*Molinia* hollows | 5-10 | 1.95 | 2 (hard to soft) | 40-50 | 5-15 | 40-50 | 20-30 | 0-5 | 10-20 | 20-30 | 0 | 0 | Shallow infilling drain (15m) | 0.9 |
| Fiddandarry | F18 | High hummocks/*Molinia* & *Sphagnum* hollows | 10-20 | 2.7 | 2 (hard to soft) | 50-60 | 5-15 | 30-40 | 20-30 | 0-5 | 10-20 | 40-50 | 0 | 0 | Shallow infilling drain (15m) | 4.3 |
| Fiddandarry | F19 | Low hummocks/*Sphagnum* pools | >30 | 3.89 | 5 (quaking) | 0-5 | 15-30 | 10-15 | 10-20 | <1 | 10-20 | 90-100 | 0 | 0 | None | 5.3 |
| Fiddandarry | F20 | Low hummock/*Molinia* hollows | 5-10 | 1.5 | 2 (hard to soft) | 60-70 | 5-15 | 10-20 | 20-30 | <1 | 10-20 | 20-30 | 0 | 0 | None | 1.5 |
| Fiddandarry | F21 | Low hummocks/*Sphagnum* lawns | 20-30 | 2.5 | 5 (quaking) | 5-10 | 30-40 | 20-30 | 10-20 | 1 | 10-20 | 80-90 | <1 | 0 | None | 3.0 |
| Fiddandarry | F22 | Low hummocks/*Sphagnum* pools | 5-10 | 3.9 | 4 (very soft to quaking) | 0 | 0 | 0-5 | 5-10 | <1 | 10-20 | 30-40 | 5-10 | <1 | Shallow (1m) drain (60m) | 4.0 |
| Fiddandarry | F23 | *Molinia* tussocks | 0 | 2.9 | 2 (hard to soft) | 90-100 | 0 | 0 | 0 | <1 | 10-20 | <1 | 0 | 0 | Shallow (0.75cm) drain (10m) | 1.4 |
| Fiddandarry | F24 | Low hummocks/*Sphagnum* lawns & pools | >30 | 4.18 | 5 (quaking) | <1 | 40-50 | 20-30 | 10-20 | 0-5 | 10-20 | 70-80 | <1 | <1 | None | 6.4 |
| Fiddandarry | F25 | Low hummocks/*Sphagnum & open* water pools | 10-20 | 2.3 | 3 (soft) | 1-5 | 25-35 | 10-20 | 10-20 | 0-5 | 10-20 | 30-40 | 5-10 | 0-5 | None | 9.5 |
| Fiddandarry | F26 | High hummocks/*Sphagnum* hollows | 10-20 | 2.08 | 2 (hard to soft) | 10-20 | 45-55 | 40-50 | 20-30 | 0-5 | 10-20 | 60-70 | 0 | 0 | Shallow infilling drain (5m). Ridges from past turf cutting evident. | 1.0 |
| Fiddandarry | F27 | Low hummocks/*Narthecium* flats | <5 | 3.79 | 3 (soft) | 0 | 20-30 | 10-15 | 5-10 | 5-10 | 5-10 | 5-10 | 10 | 0-5 | Poaching | 5.9 |
| Fiddandarry | F28 | Low hummocks/*Sphagnum* pools | 20-30 | 3.4 | 4 (very soft to quaking) | 0 | 40-50 | 10-15 | 5-10 | 5 | 5-10 | 30-40 | 5-10 | 0 | Light grazing but not obvious damage | 15.3 |
| Fiddandarry | F29 | High hummocks/*Sphagnum & open* water pools/*Narthecium* flats | 10-20 | 5 | 4 (very soft to quaking) | 0 | 30-40 | 20-30 | 20-30 | <1 | 10-20 | 30-40 | 5-10 | 0-5 | Poaching | 3.9 |
| Fiddandarry | F30 | Low hummocks/*Sphagnum* hollows/*Narthecium* flats | 5-10 | 3.24 | 2 (hard to soft) | 20-30 | 15-25 | 30-40 | 10-20 | 0-5 | 10-20 | 30-40 | 5-10 | <1 | Light grazing but not obvious damage | 3.4 |
| Garron | **G_1** | High hummocks | 10-15 | 1.20 | 2 (hard to soft) | 30-40 | 30-40 | 0-5 | 20-30 | 0 | 0 | 40-50 | 0 | 0 | Moderate grazing | 0.3 |
| Garron | **G_2** | Low hummocks | 5-10 | 2.00 | 2 (hard to soft) | 0 | 40-50 | 30-40 | 20-30 | 5-10 | 5-10 | 30-40 | 0 | 0 | None | 1.7 |
| Garron | **G_3** | High hummocks | 15 | 3.20 | 2 (hard to soft) | 30-40 | 30-40 | 0-5 | 30-40 | <1 | 5-10 | 30-40 | 0 | 0 | None | 7.3 |
| Garron | **G_4** | High hummocks | 15-20 | 1.00 | 2 (hard to soft) | <1 | 30-40 | 40-50 | 30-40 | 0-5 | 0-5 | 40-50 | 0 | 0 | None | 0.6 |
| Garron | **G_5** | Low hummocks | 0-5 | 4.47 | 2 (hard to soft) | 0 | 5-10 | 30-40 | 30-40 | 0-5 | 5-10 | 10-15 | <1 | 0 | None | 5.2 |
| Garron | **G_6** | High hummocks | 0-5 | 3.68 | 2 (hard to soft) | 90-95 | <1 | 0 | 0 | 0-5 | 10-20 | 5-10 | 0 | 0 | Drain still functional 30m away | 2.9 |
| Garron | **G_7** | Low hummocks | 20-25 | 5.55 | 4 (very soft to quaking) | 0 | 70-80 | 15-20 | 30-40 | <1 | 10-20 | 30-35 | 0 | 0 | None | 16.7 |
| Garron | **G_8** | Low hummocks | 0 | 6.20 | 2 (hard to soft) | 5-10 | 80-90 | 0 | 0 | 0-5 | 5-10 | 0-5 | <1 | 0 | None | 27.9 |
| Garron | **G_9** | Low hummocks | 0-5 | 0.90 | 1 (hard) | 10-20 | 30-40 | 10-20 | 20-30 | 0-5 | 5-10 | 10-15 | 10 | 0 | Moderate grazing | 0.2 |
| Garron | **G_10** | Low hummocks | 0-5 | 2.25 | 2 (hard to soft) | 0 | 30-40 | 20-30 | 30-40 | 0-5 | 5-10 | 20-30 | 0-5 | 0 | Light grazing | 4.4 |
| Garron | **G_11** | High hummocks | 5-10 | 1.12 | 2 (hard to soft) | 0 | 20-30 | 70-80 | 30-40 | 0-5 | 10-20 | 60-70 | 0 | 0 | None | 0.7 |
| Garron | **G_12** | Low hummocks | 0-5 | 1.05 | 1 (hard) | 99 | 0 | 10-15 | 30-40 | <1 | 10-20 | <1 | 0 | 0 | None | 0.4 |
| Garron | **G_13** | High hummocks | 5-10 | 1.60 | 2 (hard to soft) | 0 | 5-10 | 60-70 | 30-40 | <1 | <1 | 70-80 | 0 | 0 | None | 0.9 |
| Garron | **G_14** | Low hummocks | 0-5 | 5.37 | 2 (hard to soft) | 90-95 | 5-10 | <1 | 20-30 | <1 | 10-20 | 20-25 | 0 | 0 | None | 4.3 |
| Garron | **G_15** | Low hummocks | 5-10 | 3.35 | 3 (soft) | 0 | 40-50 | 30-40 | 20-30 | 0-5 | 10-20 | 30-35 | 0-5 | 0 | None | 6.9 |
| Garron | **G_16** | High hummocks | 0-5 | 1.20 | 2 (hard to soft) | 0 | 10-15 | 60-70 | 30-40 | <1 | <1 | 30-40 | 0 | 0 | None | 0.8 |
| Garron | **G_17** | High hummocks | 5-10 | 2.50 | 2 (hard to soft) | 0 | 10-15 | 70-80 | 30-40 | <1 | 20-30 | 50-60 | 0 | 0 | None | 4.4 |
| Garron | **G_18** | Low hummocks | 0-5 | 2.80 | 1 (hard) | 0 | 60-70 | 20-30 | 20-30 | 0-5 | 10-20 | 20-30 | 5-10 | 0 | None | 3.0 |
| Garron | **G_19** | Low hummocks | 0-5 | 1.90 | 2 (hard to soft) | 0 | 60-70 | 30-40 | 20-30 | 0-5 | 10-20 | 30-35 | 5-10 | 0 | None | 1.7 |
| Garron | **G_20** | Low hummocks | 0-5 | 1.00 | 2 (hard to soft) | 30-40 | 5-10 | 30-40 | 20-30 | 5-10 | 10-20 | 50-60 | 0 | 0 | None | 0.5 |
| Garron | **G_21** | Low hummocks | 0-5 | 1.10 | 1 (hard) | 90-95 | <1 | 10-20 | 30-40 | <1 | 20-30 | 5-10 | 0 | 0 | None | 0.9 |
| Letterunshin | **L_1** | Low hummocks and bare peat/ *Narthecium* flats | <1.0 | 3 | 2 (hard to soft) | <1 | 15-25 | 10-20 | 20-30 | 10-20 | 5-10 | 10-15 | 10-20 | <1 | Light grazing | 6.8 |
| Letterunshin | **L_2** | Low hummocks | 0-5 | 2.65 | 2 (hard to soft) | 5-10 | 0 | 10-20 | 20-30 | 10-20 | 5-10 | 10-20 | 5-10 | 0 | Light grazing | 1.5 |
| Letterunshin | **L_3** | Low hummocks | 0-5 | >3.0 | 2 (hard to soft) | 10-20 | 0 | 10-20 | 20-30 | 10-20 | 5-10 | 20-25 | <1 | 0 | Light grazing | 0.8 |
| Letterunshin | **L_4** | High hummocks | 0-5 | 1.6 | 1 (hard) | 10-20 | 0 | 10-20 | 20-30 | 5-10 | 5-10 | 25-30 | 0-5 | 0 | Moderate grazing | 0.3 |
| Letterunshin | **L_5** | High hummocks | 0-5 | >3.0 | 1 (hard) | 40-50 | 5-15 | 20-30 | 20-30 | 5-10 | 5-10 | 30-40 | <1 | 0 | Light grazing | 2.6 |
| Letterunshin | **L_6** | High hummocks | 0-5 | 1.2 | 1 (hard) | 40-50 | 0 | 40-50 | 30-40 | 0-5 | 20-30 | 10-15 | 0 | 0 | 45 | 0.5 |
| Letterunshin | **L_7** | Low hummocks | 0-5 | 1.6 | 2 (hard to soft) | 60-70 | 5-15 | 20-30 | 20-30 | 5-10 | 5-10 | 30-40 | 0 | 0 | Light grazing | 0.3 |
| Letterunshin | **L_8** | Low hummocks and *Sphagnum* lawns | 10-15 | >3.0 | 4 (very soft to quaking) | 0-5 | 0 | 10-20 | 10-20 | 5-10 | 5-10 | 70-80 | <1 | <1 | None | 2.1 |
| Letterunshin | **L_9** | Low hummocks and *Sphagnum* lawns | 20-30 | >3.0 | 5 (quaking) | <1 | 0 | 10-20 | 10-20 | 5-10 | 5-10 | 50-60 | 5-10 | 0 | None | 3.5 |
| Letterunshin | **L_10** | Low hummocks | 5-10 | >3.0 | 2 (hard to soft) | 10-20 | n/a | 20-30 | 20-30 | 5-10 | 5-10 | 20-30 | <1 | n/a | Light grazing | 1.2 |
| Letterunshin | **L_11** | Low hummocks and *Sphagnum* pools | 20-30 | >3.0 | 5 (quaking) | <1 | n/a | 20-30 | 20-30 | 5-10 | 5-10 | 80-90 | <1 | n/a | None | 1.5 |
| Letterunshin | **L_12** | Low hummocks and *Sphagnum* pools | 20-30 | >3.0 | 5 (quaking) | <1 | n/a | 20-30 | 20-30 | 0-5 | 5-10 | 90-100 | <1 | n/a | None | 4.5 |
| Letterunshin | **L_13** | Low hummocks | 0-5 | 3 | 2 (hard to soft) | <1 | n/a | 30-40 | 20-30 | 5-10 | 5-10 | 20-30 | 0-5 | n/a | None | 1.7 |
| Letterunshin | **L_14** | Low hummocks | 0-5 | 1.95 | 2 (hard to soft) | 50-60 | n/a | 20-30 | 10-20 | 5-10 | 5-10 | 30-40 | <1 | n/a | None | 0.6 |
| Letterunshin | **L_15** | Low hummocks | 0-5 | 2.05 | 1 (hard) | 70-80 | n/a | 10-20 | 20-30 | 0-5 | 5-10 | 10-15 | <1 | n/a | Evidence of old burning | 0.9 |
| Letterunshin | **L_16** | High hummocks and open water pools | 5-10 | >3.0 | 2 (hard to soft) | 0 | n/a | 30-40 | 10-20 | 5-10 | 5-10 | 10-20 | 20-25 | n/a | Light grazing and evidence of burning. | 11.6 |
| Letterunshin | **L_17** | Low hummocks and *Sphagnum* hollows | 20-25 | >3.0 | 4 (very soft to quaking) | 0-5 | n/a | 30-40 | 10-20 | 5-10 | 5-10 | 50-60 | <1 | n/a | Light grazing | 2.2 |
| Letterunshin | **L_21** | Low hummocks and *Narthecium*/bare peat flats | 5-10 | >3.0 | 4 (very soft to quaking) | 0 | n/a | 40-50 | 20-30 | 5-10 | 5-10 | 30-35 | 10-20 | n/a | None | 7.2 |
| Letterunshin | **L_22** | Low hummocks and *Narthecium*/bare peat flats | 5-10 | >3.0 | 4 (very soft to quaking) | 0 | n/a | 10-20 | 20-30 | 10-20 | 5-10 | 20-30 | 5-10 | n/a | None | 8.2 |
| Letterunshin | **L_4A** | High hummocks | 0-5 | 1.35 | 1 (hard) | 40-50 | n/a | 20-30 | 30-40 | <1 | 20-30 | 10-15 | 0 | n/a | Light grazing | 0.2 |
